# Formulation of pH-Responsive Quatsomes from Quaternary Bicephalic Surfactants and Cholesterol for Enhanced Delivery of Vancomycin against Methicillin Resistant *Staphylococcus aureus*

**DOI:** 10.3390/pharmaceutics12111093

**Published:** 2020-11-14

**Authors:** Daniel Hassan, Calvin A. Omolo, Victoria Oluwaseun Fasiku, Ahmed A Elrashedy, Chunderika Mocktar, Bongani Nkambule, Mahmoud E. S. Soliman, Thirumala Govender

**Affiliations:** 1 Discipline of Pharmaceutical Sciences, College of Health Sciences, University of KwaZulu-Natal, Private Bag X54001, Durban 4000, South Africa; daniel4hassanuk1@yahoo.com (D.H.); victachriss@gmail.com (V.O.F.); ahmedelrashedy45@gmail.com (A.A.E.); Mocktarc@ukzn.ac.za (C.M.); Soliman@ukzn.ac.za (M.E.S.S.); 2 Department of Pharmaceutics and Pharmacy Practice, School of Pharmacy and Health Sciences, United States International University-Africa, P. O. Box 14634, Nairobi 00800, Kenya; 3 Department of Physiology, School of Laboratory Medicine and Medical Sciences, College of Health Sciences, University of KwaZulu-Natal, Private Bag X54001, Durban 4000, South Africa; Nkambuleb@ukzn.ac.za

**Keywords:** quatsomes, pH-responsive, vancomycin, methicillin-resistant *Staphylococcus aureus* (MRSA), quaternary, bicephalic surfactant

## Abstract

Globally, human beings continue to be at high risk of infectious diseases caused by methicillin-resistant *Staphylococcus aureus* (MRSA); and current treatments are being depleted due to antimicrobial resistance. Therefore, the synthesis and formulation of novel materials is essential for combating antimicrobial resistance. The study aimed to synthesize a quaternary bicephalic surfactant (StBAclm) and thereof to formulate pH-responsive vancomycin (VCM)-loaded quatsomes to enhance the activity of the antibiotic against MRSA. The surfactant structure was confirmed using ^1^H, ^13^C nuclear magnetic resonance (NMR), Fourier-transform infrared spectroscopy (FT-IR), and high-resolution mass spectrometry (HRMS). The quatsomes were prepared using a sonication/dispersion method and were characterized using various in vitro, in vivo, and in silico techniques. The in vitro cell biocompatibility studies of the surfactant and pH-responsive vancomycin-loaded quatsomes (VCM-StBAclm-Qt_1_) revealed that they are biosafe. The prepared quatsomes had a mean hydrodynamic diameter (MHD), polydispersity index (PDI), and drug encapsulation efficiency (DEE) of 122.9 ± 3.78 nm, 0.169 ± 0.02 mV, and 52.22 ± 8.4%, respectively, with surface charge switching from negative to positive at pH 7.4 and pH 6.0, respectively. High-resolution transmission electron microscopy (HR-TEM) characterization of the quatsomes showed spherical vesicles with MHD similar to the one obtained from the zeta-sizer. The in vitro drug release of VCM from the quatsomes was faster at pH 6.0 compared to pH 7.4. The minimum inhibitory concentration (MIC) of the drug loaded quatsomes against MRSA was 32-fold and 8-fold lower at pH 6.0 and pH 7.4, respectively, compared to bare VCM, demonstrating the pH-responsiveness of the quatsomes and the enhanced activity of VCM at acidic pH. The drug-loaded quatsomes demonstrated higher electrical conductivity and a decrease in protein and deoxyribonucleic acid (DNA) concentrations as compared to the bare drug. This confirmed greater MRSA membrane damage, compared to treatment with bare VCM. The flow cytometry study showed that the drug-loaded quatsomes had a similar bactericidal killing effect on MRSA despite a lower (8-fold) VCM concentration when compared to the bare VCM. Fluorescence microscopy revealed the ability of the drug-loaded quatsomes to eradicate MRSA biofilms. The in vivo studies in a skin infection mice model showed that groups treated with VCM-loaded quatsomes had a 13-fold decrease in MRSA CFUs when compared to the bare VCM treated groups. This study confirmed the potential of pH-responsive VCM-StBAclm quatsomes as an effective delivery system for targeted delivery and for enhancing the activity of antibiotics.

## Highlights

- A novel surfactant (StBAclm) was synthesized, and its structure confirmed.
- Vancomycin-loaded pH-responsive quatsomes (VCM-StBAclm-Qt1) were prepared from StBAclm.
- The in vitro drug results showed a faster VCM release from the quatsomes at pH 6.0 compared to pH 7.4 and enhanced in vitro antibacterial activity against MRSA as compared to bare VCM.
- There was an enhanced bacterial killing kinetics and high perforation of MRSA membrane cell wall by the quatsomes.
- A higher electrical conductivity, reduced DNA, and protein concentration were achieved by the quatsomes.
- There was an enhanced in vivo antibacterial activity of the drug quatsomes against MRSA compared to bare VCM in a mice skin infection model.

## 1. Introduction

Novel drug delivery systems using nanotechnology are an alternative option for solving antimicrobial resistance. Nanosized drug delivery systems offer greater versatility due to size, composition, stimuli-responsiveness [1], biocompatibility, biodegradability, reduced side effects [2,3], and decreased exposure of antibiotics to healthy sites when compared to conventional dosage forms [4]. Loading of antibiotics into nanosized drug delivery systems offers higher drug pharmacokinetic profiles, enhanced antibacterial activity, and protects the drug from enzymatic destruction leading to fewer side effects [5,6]. These traits tend to address the limitations of conventional dosage forms, which have been used for treating infectious diseases since the introduction of antibiotics [7,8].

Several nanodrug delivery systems have been involved in the effective delivery of antimicrobials with great success, and currently, they are being been introduced into the market [9,10]. Lipidic vesicles are the most-employed nanostructures for drug delivery since their serendipitous discovery in 1964 [11]. They can entrap hydrophobic molecules within their bilayers and hydrophilic molecules in their lumen, which makes them excellent nanocarriers for the delivery of drugs [12,13]. Despite their desirable qualities, their use in drug delivery has been limited by their low degree of structural homogeneity, which is a critical requirement for their enhancement of the pharmacological property of loaded drugs [14]. Their stability is also a limiting factor because their lipid building blocks are insoluble in water [15,16]. These limitations have prompted research into other vesicles for drug delivery, such as polymersomes from amphiphilic polymers [17], dendrimersomes from dendrimers [18,19], and peptosomes from self-assembling peptides [20]. However, these vehicles still have limitations due to their structural stability, low entrapment, membrane thickness, and area density, which is greatly affected by block length and the size of the polymer, dendrimers, or peptides [21].

The search for superior vehicles for drug delivery has resulted in the development of quatsomes. They are fairly new nanoscopic vesicles, composed of sterols and quaternary ammonium surfactants in defined molar ratios [13]. Unlike conventional liposomes, they have been reported to be stable for a long storage time, have high vesicle-to-vesicle homogeneity in size and lamellarity and high drug-loading capacity [22]. Furthermore, quatsomes have been shown to offer protection against premature drug degradation; they have intracellular penetration [23]; and they can be a potential platform for site-specific delivery of drugs. Their described desirable qualities and their inherent antimicrobial activity make them suitable candidates for antibiotic delivery [13,23]. However, so far studies in the literature have reported on quatsomes with the aim of improving the toxicity profile of cationic compounds employed to formulate them, understanding the physical–chemical properties of their membrane [22,24] and identifying their potential as antibiofilm agents [13,24] due to their cationicity.

The reported quatsomes have mostly been prepared from cationic quaternary surfactants available in the market. Most of these surfactants are toxic to various cells in the body and due to that, their systemic toxicity application is limited [24,25,26]. The antibacterial activity of the quaternary ammonium compounds (QAC) has been attributed to a higher positive charge density of the biomaterials [27]. However, a higher positive charge density of the QAC often leads to the toxicity of these compounds [28]. Therefore, to formulate quatsomes that can be efficient for drug delivery, new QAC biomaterials with a balance between the cationic effect that causes inherent antimicrobial activity and biosafety for systemic use need to be developed. Therefore, this study reports for the first time a quaternary ammonium surfactant whose charge is pH-dependent for the formulation of quatsomes for the delivery of antibiotics. We envisage that such a surfactant will be able to avoid the safety concerns associated with conventional cationic surfactants and at the same time potentiate the antibacterial activity of the antibiotics being delivered.

Recently, pH-responsive drug delivery systems for antibiotics have been receiving interest since they can respond to the unique acidic conditions at infection sites and offer increased drug delivery and receptor binding to improve treatment. Hence, this study reports, also for the first time, a pH responsive quatsome coined from a novel pH-responsive quaternary bicephalic surfactant (StBAclm) and cholesterol (CHol). We envisage a biosafe, self-assembled quatsome to occur due to the electrostatic interactions between the hydrophobic tail of the surfactant and CHol; whilst the pH-responsiveness will be due to the protonation and deprotonation of the bicephalic carboxylic arms of the surfactant. Moreover, the quatsome’s inherent antimicrobial activity will potentiate the antibacterial activity of the loaded antibiotics. In addition, the reduced positive charge density of the quaternary surfactant due to pH-responsive surface charge switching will make the system more biosafe. The succinct in vitro, in vivo, and in-silico evaluation of this novel pH-responsive quatsome is reported in this study.

## 2. Materials and Methods

### 2.1. Materials

Stearylamine, *tert*-butyl acrylate (tBA), methanol (MeOH), trifluoroacetic acid (TFA), triisopropylsilane (TIPs), methyl iodide (MI), cholesterol (CHol), and silica gel were purchased from Sigma-Aldrich (St. Louis, MO, USA). Vancomycin (VCM) and MTT were purchased from Sinobright Import and Export Co., Ltd. (Shenzhen, China) and Merck Chemicals (Darmstadt, Germany), respectively. Furthermore, sheep’s blood was obtained from Polychem, Durban, South Africa, while all other chemicals and solvents used were of analytical grade. NB, MHB, MHA, were obtained from Biolab (Gauteng, South Africa). Methicillin-resistant *Staphylococcus aureus* (MRSA; *S. aureus* Rosenbach ATCC BAA 1683), Dulbecco′s modified Eagle′s medium (DMEM) and fetal bovine serum (FBS) were obtained from Life Technologies (Austin, TX, USA), and penicillin-streptomycin (pen/strep) was purchased from Lonza (Portsmouth, NH, USA). Propidium iodide (PI) and Syto9 dye kits were obtained from Thermofisher (Eugene, OR, USA). Bacteria DNA and BCA Kits were purchased from Zymo Research California, (Tustin, CA, USA), and Wako Chemicals (Richmond, VA, USA), respectively. Millipore water used during the study was purified in the laboratory using the Milli-Q water purification system (Millipore corp., Boston, MA, USA). In vivo studies were conducted following a University of KwaZulu-Natal’s (UKZN) Animal Research Ethics Committee approval on 15 April 2019 (Approval number: AREC/104/015PD).

### 2.2. Instrumentation (^1^H NMR, ^13^C NMR, FT-IR and HR-MS Spectra)

^1^H NMR, ^13^C NMR and FT-IR spectra were conducted and recorded on a Bruker 400 Ultra shield™ (Durham, United Kingdom), a NMR spectrometer and a Bruker Alpha-p spectrometer with a diamond ATR (Leipzig, Germany), respectively. HR-MS was accomplished on a Water Micromass LCT Premier TOF-MS (Wilmslow, United Kingdom). The system (GPC: TFA) consisted of a Water 717 plus auto-sampler, a Water 1515 isocratic HPLC pump, a Waters refractive index detector, and a Water 600E system controller. Optical density (OD) was measured on Spectrostar Nano, BMG Labtech (Ortenberg, Germany).

### 2.3. Synthesis and Characterization of the Surfactant

The bicephalic surfactant was synthesized as per Scheme 1. Characterization and structural elucidation is found in the Supplementary Materials Figures S1–S13.

### 2.4. Formulation of VCM-Loaded Quatsomes (VCM-StBAclm-Qt)

The quatsomes were prepared according to a previously reported method with slight modification [22]. Briefly, StBAclm (final StBAclm concentration 0.5, 1, 2.5, 5, and 10 mg/mL) and CHol were accurately weighed into beakers keeping the molar ratio of the two excipients constant (1:1). Drug-loaded quatsomes were formulated by the addition of the bicephalic surfactant, VCM, and CHol at different concentrations. This was followed by the addition of 40 mL of Milli-Q water, and the mixture was further sonicated for 10, 15, 20, and 30 min (30/10 s on/off cycle, 30% amplitude) using a probe-sonicator in an ice bath. The dispersions were further stirred at 500 rpm on a magnetic stirring plate for 24 h at room temperature. Milli-Q water was used as the dispersion medium to avoid changes in the solubility of StBAclm as the presence of electrolytes can adversely affect the formation of the quatsomes [13,22]. After 24 h of equilibration, potential aggregates were removed from the blank (StBAclm-Qt) and the drug-loaded VCM-StBAclm-Qt quatsomes by filtration using a syringe/filter (0.45 μm pore size).

### 2.5. Characterization of the VCM-StBAclm-Qt Quatsomes

#### 2.5.1. Mean Hydrodynamic Diameter (MHD), Polydispersity Index (PDI), Zeta Potential (*ζ*), and Morphology

The mean hydrodynamic diameter (MHD), polydispersity index (PDI), and *ζ* of VCM-StBAclm-Qt quatsomes were analyzed using a Zetasizer Nano ZS90 (Malvern Instruments Ltd., Malvern, UK). Cryogenic-HR transmission electron microscopy (Cryo-HR-TEM) on a Jeol, JEM-2100 (Tokyo, Japan) was used to examine the morphology of VCM-StBAclm-Qt quatsomes. Briefly, the drug-loaded quatsomes were diluted accordingly and mounted onto the copper grid surface, and immediately 2% uranyl acetate (UA) solution was used to stain. The excess VCM-StBAclm-Qt quatsomes and stain on the grid were removed by blotting off with filter paper and affixed with liquid nitrogen [29]. Before the visualization of the quatsomes’ morphology, the sample grid was kept in liquid nitrogen and transferred into the chamber. The images were captured and obtained at an accelerating voltage of 200 kV.

#### 2.5.2. Drug Entrapment Efficiency (DEE%) and Drug Loading Capacity (DLC%)

The VCM-StBAclm-Qt quatsomes were further characterized to determine drug entrapment efficiency (DEE%) and drug loading capacity (DLC%). The DEE % was determined by an ultrafiltration method, as previously reported using the regression equation y = 0.0038x − 0.0031, with a linear regression coefficient (R²) of 0.9998 [30,31] and the details can be found in the supplementary materials.

### 2.6. Molecular Modeling Simulations (MDS) 

#### 2.6.1. Vancomycin, CHol, and Surfactant Simulation Quatsomes

To evaluate the self-assembling quatsomes and the encapsulation of VCM, molecular modeling simulations were employed. The VCM, CHol, and the surfactant (StBAclm) structures were drawn with ChemDraw professional 17.0. A Chimera molecular modeling suite was used to prepare the initial VCM-CHol-surfactant complex [32]. The LEaP (link, edit, and parm) module implemented in AMBER 14 was used to combine, neutralize, and solvate the system by adding hydrogen atoms, chloride, and sodium ions and suspending them in an orthorhombic box of TIP3P water molecules, such that all atoms were within 10 A of the box edges [33]. The system contained a total of 2082 water molecules and 1 molecule of VCM, 1 molecule of CHol, and 1 molecule of the surfactant. The system was minimized for 2500 steps with a strong constraint on VCM, CHol, and surfactant for 1000 steps (500 steepest descent followed by 500 steps of the conjugate gradient). Using a collision frequency of 1.0 ps-1, harmonic restraint of 5 kcal/mol Å on the solutes and gradual heating of the systems up to 300 K using the Langevin thermostat, the system underwent a further 1000 steps of full minimization in the canonical ensemble for 50 ps. Via NPT(constant Number, Pressure and Temperature) ensemble, 50 ps of density equilibration was undertaken at 500 ps, 300 K temperature, 1 bar pressure, and a coupling constant of 2 ps. Molecular modeling simulations (MDS) was then performed for 10 ns using classical MDS with a time step of 2 fs. Frames were recorded after every 500 steps of the simulation. A SHAKE algorithm [34] was used to constrain the bond lengths involving hydrogen atoms.

#### 2.6.2. VCM, CHol, and Surfactant Self-Assembly Complexation Simulation 

The Chimera tool was used for random insertion of six complexes of VCM, CHol, and the surfactant complex. The system was then minimized for 2500 steps with strong constraints on VCM, cholesterol, and the surfactant for 1000 steps (500 steepest descent followed by 500 steps of the conjugate gradient). This was followed by 1000 steps of full minimization Langevin thermostat, with a collision frequency of 1.0 ps-1 with a harmonic restraint of 5 kcal/mol Å on the solutes. At the same time, there was a gradual heating up of the systems to a temperature of 300.00 K in the canonical ensemble for 50 ps. Using NPT ensemble, an additional 50 ps of density equilibration was performed, this was followed by a final 500 ps equilibration at 300 K, 1 bar pressure, and a coupling constant of 2 ps, before a 10 ns MDS production via classical MDS with a time step of 2 fs. After 500 steps of simulation the frames were recorded and all the hydrogen bond atoms were constrained using a SHAKE algorithm [34]. All the molecular dynamics simulations were carried out using the GPU Amber 14 software package [35].

#### 2.6.3. Post-Dynamic Analysis and Binding-Free Energy Calculations

The trajectories generated after MDS simulations were each saved every 1 ps, followed by analysis using the CPPTRAJ [36] module employed in AMBER 14 suit. All plots and visualizations were completed using the Origin data analysis tool [37] and Chimera [32], respectively. Binding-free energy calculation is an important endpoint method that elucidates the mechanism of binding between a ligand and protein, including both enthalpic and entropic contributions [38,39]. To estimate the binding affinity of the docked systems, the free binding energy was calculated using the molecular mechanics/GB surface area method (MM/GBSA) [40]. Binding-free energy was averaged over 1000 snapshots extracted from the 10 ns trajectory. The free binding energy has been computed by this method for each molecular species (complex, ligand, and receptor) and can be represented as (Equations (1)–(5)):

(1) $$\Delta G_{\mathrm{bind}}=G_{\mathrm{complex}}-G_{\mathrm{receptor}}-G_{\mathrm{ligand}}$$

(2) $$\Delta G_{\mathrm{bind}}=E_{\mathrm{gas}}+G_{\mathrm{sol}}-\mathrm{TS}$$

(3) $$E_{\mathrm{gas}}=E_{\mathrm{int}}+E_{\mathrm{vdw}}+E_{\mathrm{ele}}$$

(4) $$G_{\mathrm{sol}}=G_{\mathrm{GB}}+G_{\mathrm{SA}}$$

(5) $$G_{\mathrm{SA}}=\gamma \mathrm{SASA}$$

The terms E_gas_, E_int_, E_ele_, and E_vdw_ symbolize the gas-phase energy, internal energy, Coulomb energy, and van der Waals energy, respectively. The E_gas_ was directly assessed from the force field terms. Solvation free energy (G_sol_) was assessed from the energy involvement from the polar states (G_GB_) and non-polar states (G). The non-polar solvation energy (G_SA_) was determined from the solvent-accessible surface area (SASA) using a water probe radius of 1.4 Å; while the polar solvation (G_GB_) contribution was assessed by solving the GB equation. S and T symbolize the total entropy of the solute and temperature, respectively [40].

### 2.7. In Vitro Hemolytic Activity Assay of the VCM-StBAclm-Qt1 Quatsomes

The hemolytic activity assay to assess the hemolytic behavior of the VCM-StBAclm-Qt1 quatsomes was performed according to a previously reported method [41,42]. Fresh whole sheep blood (100 μL) was added to a freshly prepared PBS pH 7.4 (1 mL) containing 50, 100, 150, 200, and 250 µg of VCM-loaded StBAclm-Qt1 quatsomes. The mixture was incubated at 37 °C for 1 h. For the negative and positive controls, 100 μL of the sheep’s blood was added to the 900 μL of PBS and distilled water, respectively. Immediately after one hour’s incubation, the solutions were centrifuged at 10,000 rpm for exactly five minutes. This was followed by pipetting 200 μL of the supernatant and seeding into 96-well plates for hemoglobin release at 540 nm using a microplate spectrophotometer (Spectrostar Nano, Ortenberg, Germany; *n* = 4). The Equation (6), below, where As, An, and Ap represent sample absorbance and negative and positive controls, respectively, was used to calculate the percentage hemolysis.

(6) $$\frac{\left(\mathrm{As}\;-\mathrm{An}\right)}{\left(\mathrm{Ap}\;-\mathrm{An}\right)}\;\times 100\%$$

### 2.8. In Vitro Cytotoxicity of StBAclm and VCM-StBAclm-Qt against Different Cell Lines

The biocompatibility of the novel StBAclm and VCM-StBAclm-Qt quatsomes was evaluated using a modified MTT assay method previously reported [10,43]. Four different cell lines were used, viz: human embryonic kidney (HEK-293); liver hepatocellular carcinoma (Hep-G2); human breast adenocarcinoma (MCF-7); and adenocarcinomic alveolar basal epithelial cells (A-549) were obtained from our own storage and repropagated. Briefly, all four cell lines were grown exponentially in DMEM media supplemented with 10% FBS (*v/v*) and 1% Pen-Strep (*v/v*) at 37 °C in a 5% CO_2_ humidified atmosphere. The cell lines containing greater than 80% confluency were trypsinized and seeded into 96-well plates with 5 × 10^3^ density, and then incubated for 24 h. The StBAclm and VCM-StBAclm-Qt quatsomes were prepared and diluted with sterilized Milli-Q water separately, to a final concentration of 20, 40, 60, 80, 100, and 120 μg/mL of VCM in the formulation [10]. Subsequently, the culture medium and the quatsomes were removed after 48 h and replaced with 100 μL of fresh medium followed by the addition of MTT solution (5 mg/mL in PBS) in each well. The cells were further incubated for an additional four hours, followed by the removal of the solution previously added. Afterwards, solubilization of the MTT formazan was achieved by the addition of 100 μL DMSO into each well. The optical density of each well was measured on a microplate spectrophotometer (Spectrostar Nano, Ortenberg, Germany) at 540 nm. All the experiments were performed in six replicates and percentage cell viability was calculated as follows (Equation (7)).

(7) $$\%\;\mathrm{Cell}\;\mathrm{viability}=\left(\frac{A540\;\mathrm{nm}\;\mathrm{treated}\;\mathrm{cells}}{A540\;\mathrm{nm}\;\mathrm{untreated}\;\mathrm{cells}}\right)\times \;100$$

### 2.9. In Vitro Drug Release of VCM from VCM-loaded StBAclm-Qt Quatsomes

The drug release of VCM was performed as per previously reported procedures [44,45]. Two milliliters of the bare VCM, VCM-StBAclm-Qt, and the corresponding blank StBAclm-Qt were loaded into the dialysis bags with a size porosity of 8000–14,400 Da. The dialysis bags containing the bare VCM, drug-loaded quatsomes, and the corresponding blank were placed separately in a 40 mL receiver compartment of PBS (pH 6.0 and pH 7.4) at 37 °C in a shaking incubator at 100 rpm. Samples of 3 mL were withdrawn at specific time intervals from the receiver compartment solution. Immediately, an equal amount of fresh PBS (pH 6.0 and pH 7.4) was replaced into the corresponding compartment to keep the initial receiver volume constant. The quantity of VCM released from the dialysis bags in the receiver chamber was measured spectrophotometrically with Shimadzu UV 1601, Japan, at 280 nm, using the regression equation mentioned in Section 2.5.2. The release fraction of VCM from VCM-StBAclm-Qt quatsomes and VCM solution was calculated using the Equation (8) below *(n* = 3); where At is the amount of VCM released from drug-loaded VCM-StBAclm-Qt_1_ quatsomes at time t, and Ap the amount of VCM preloaded in the VCM-StBAclm-Qt_1_ quatsomes.

(8) $$\%\;\mathrm{Cumulative}\;\mathrm{release}\;\mathrm{profile}\;=\left(\frac{\mathrm{At}}{\mathrm{Ap}}\right)\times \;100\%$$

### 2.10. Evaluation of In Vitro Antibacterial Activities on VCM-StBAclm-Qt Quatsomes

#### 2.10.1. Determination of Minimum Inhibitory Concentrations (MICs)

The MICs studies were conducted against MRSA, according to the previously reported procedure [46]. The bacteria were cultured and grown in MHB with relevant dilutions achieved at 5 × 10^5^ colony forming units per mL (CFU/mL) [47]. The bare VCM, StBAclm-Qt, and VCMwere serially diluted in MHB and incubated with bacteria culture (5 × 10^5^) colony forming units per milliliter (CFU/mL) for exactly 20 h in a shaking incubator at 37 °C and 100 rpm. Precisely, 10 μL of the serially diluted solutions was spotted on MHA plates and incubated for 24 h; and the MIC was regarded as the point with no visible bacteria growth. The combined antimicrobial effect of blank quatsomes and VCM in the VCM StBAclm-Qt quatsomes against MRSA was assessed using the FIC method [48]. The FIC index was calculated using the equations illustrated in Section S6 in the supplementary material.

#### 2.10.2. Time Killing Assays VCM-StBAclm-Qt Quatsomes 

An overnight MRSA cultured in nutrient broth (NB) was diluted with phosphate buffer saline (pH 7.4) to a concentration (5 × 10^5^ CFU/mL). Bare VCM and VCM-StBAclm-Qt quatsomes were added at concentrations equivalent to 5 × MIC. For the negative control, sterile deionized water was added into the bacterial MHB alone, and bacterial cell viability was observed for up to 24 h. Tested materials, bacteria alone, bare VCM and VCM-StBAclm-Qt quatsomes were removed at designated times, serially diluted in PBS, plated on an MHA plate, and incubated for 24 h at 37 °C. After 24 hour’s incubation, CFU was counted, converted to log_10_ values and plotted on a graph as seen in Equation (9) [49].

(9) $$\%\;W=\left(\frac{\mathrm{Population}\;\mathrm{of}\;\mathrm{control}\;\mathrm{bacteria}-\;\mathrm{population}\;\mathrm{of}\;\mathrm{experimental}\;\mathrm{bacteria}}{\mathrm{Population}\;\mathrm{of}\;\mathrm{control}\;\mathrm{bacteria}}\right)\times \;100$$

### 2.11. Molecular and Mechanistic Studies on VCM-StBAclm-Qt Quatsomes 

#### 2.11.1. Effect of VCM-StBAclm-Qt Quatsomes on Electrical Conductivity, DNA, and Protein Leakage

The electrical conductivity and DNA and protein leakage studies were performed as per previously reported methods [50] and details can be found in the supplementary material, Section S7.

#### 2.11.2. Bacterial Membrane Disruption

The bacterial membrane disruption by the quatsomes was carried out using suspensions of MRSA with a concentration of 1.5 × 10^8^ CFU/mL in PBS (pH 7.4). The bacteria were incubated with VCM-StBAclm-Qt quatsomes containing 153 μg/mL of VCM (50:50) ratio for four hours in a sterilized eppendorf tube. The samples were appropriately diluted before being mounted on the copper grid as per a previously reported method [51]. The excess sample was drawn off by blotting with filter paper and allowed to dry at 25 °C before measurement. The images of the samples were obtained using a high-resolution transmission electron microscope (brightfield, darkfield, TEM-JEOL HR-TEM 2100). 

#### 2.11.3. Fluorescence-Activated Cell Sorting (FACS) Bacterial Cell Viability

The bacterial cell viability was assessed using a previously reported flow cytometry method [52]. Briefly, MHB was used to grow pure MRSA culture overnight at 100 rpm in a shaking incubator (Labcon, Petaluma, CA, USA) at 37 °C. Serial dilution of the bacteria at final concentrations of CFU/mL of 5 × 10^5^ was used. Of the bacteria suspension 15 μL was pipetted into the 96-well plate containing 135 μL of bare VCM at MIC concentrations of 31.25 μg/mL; and VCM-StBAclm-Qt quatsomes at MIC concentration of 3.90 μg/mL. The mixture was incubated for an additional six hours at 37 °C and 100 rpm. The percentage cell viability was determined after six hours of incubation, and untreated MRSA cells were used as the negative control [53]. Exactly 50 μL of the bare VCM and VCM-StBAclm-Qt quatsomes broths were appended into the flow cytometry tubes containing 350 μL of sheath fluid, vortexed, and incubated for approximately five minutes [53,54]. The cytometry tubes containing the samples were incubated for another 30 min with 5 μL 0.1% *v/v* of PI obtained from 50 μg/mL the stock solution and cell-permeant dyes 0.1% *v/v* of SYTO^®^ 9 obtained from 3.34 mM stock solution respectively. The PI fluorescence was excited at a 455 nm laser and gathered between 636 nm bandpass filter; while the Syto9 excitation laser was set at a 485 nm laser and collected at a 498 nm bandpass filter [55,56,57]. The bacteria were gated using a forward scatter (FSC), with approximately 10,000 cells gathered for individual samples and this was done in triplicate. Particles smaller than the bacteria were detected at a threshold of 100 in the SSC analyses to avoid background signals [58]. Data were captured from the flow cytometry and analyzed using Kaluza-1.5.20 software (Beckman Coulter, Indianapolis, IN, USA) [56,59].

#### 2.11.4. Reduction of MRSA Biofilm using Fluorescence Microscopy

MRSA biofilm eradication by bare VCM and VCM-StBAclm-Qt quatsomes was quantified using fluorescence microscopy, according to a previously reported procedure [60]. Briefly, coverslips were placed in a 24-well plate. Exactly 4 mL of MBH was added into the 24-well, plate followed by the addition of 2 mL of MRSA (1.5 × 10^8^ CFU/mL) suspensions grown in MHB. The solution was incubated for four days at 37 °C in a pH 7.4 buffer to form matured biofilm. Before treatment, the media used to grow the bacteria were removed from the 24-well plates using a sterilized Pasteur pipette to avoid any contamination. The plate wells were subsequently washed four times using sterilized water to remove excess and non-adhered bacteria. Exactly 1 mL of bare VCM suspension and VCM-StBAclm-Qt1 quatsomes, each containing 125 μg/mL of VCM, were added into the appropriate 24-wells plates and incubated for an additional twelve hours at 37 °C. After incubation, the wells were washed with PBS (pH 7.4) to remove the non-adhered treatments on the biofilms. Coverslips were stained with 30 μL of Syto9 and propidium iodide (PI) dyes in deionized water and left in the dark to incubate for another 30 min. The treated and stained 24-well plates were washed for the second time to remove excess dyes, and the cover plate was carefully glued and inverted on a microscope glass slide. Biofilm reduction by bare VCM and the VCM-StBAclm-Qt quatsomes were visualized on a fluorescence microscope (Nikon Eclipse 80i FM, Shinagawa, Japan).

### 2.12. In Vivo Antibacterial Activity

For proof of concept of enhanced antibacterial activity, an in vivo antibacterial activity study of the bare VCM and VCM-StBAclm-Qt quatsomes, was conducted following a University of KwaZulu-Natal’s (UKZN) Animal Research Ethics Committee approved mice skin infection model (Approval number: AREC/104/015PD) [61,62]. The humane use of animals was achieved using the guidelines of the UKZN AREC and the South African National Standard SANS 10386:2008. Male BALB/c mice of approximately 18–20 g were obtained from the Biomedical Research Unit (BRU), of UKZN. The mice were then divided into treatment and positive and negative control groups (n = 4). The mice skin, which was the site of administration of the treatments, was shaved and disinfected using 70% ethanol 24 h before the experiment. The following day, 50 µL of MRSA (1.5 × 10^8^ CFU/mL), suspended in saline, was injected intradermally. Thirty minutes after bacterial infection, 50 µL of the bare VCM, VCM-StBAclm-Qt quatsomes, and saline were injected separately at the same site of infection to the various test groups. The mice were kept under observation for 48 h with normal 12 h of light and dark conditions, at 19–23 °C, and 55% ± 10% relative humidity with adequate ventilation. After hours, the mice were euthanised with halothane and the infected area of the skin was harvested and homogenized in 5 mL PBS of pH 7.4. Tissue homogenates were serially diluted in the PBS; 50 µL of the homogenized PBS was spotted on nutrient agar plates and incubated at 37 °C for 24 h. The number of CFU and the CFU/mL were counted and calculated using the Equation (10) below:

(10) $$\mathrm{CFU}/\mathrm{mL}\;=\frac{\mathrm{number}\;\mathrm{of}\;\mathrm{colonies}\;x\;\mathrm{dilution}\;\mathrm{factor}}{\mathrm{volume}\;\mathrm{of}\;a\;\mathrm{culture}\;\mathrm{plate}}$$

### 2.13. Stability Studies

To study the stability of the novel drug-loaded quatsomes (VCM-StBAclm-Qt), the samples were sealed in a 50 mL vial in a pH 7.4 phosphate buffer in the dark, after flushing with nitrogen, and were stored at 4 °C and 25 ± 1 °C, respectively, for 30, 60, and 90 days. The stability of the VCM-StBAclm-Qt quatsomes was assessed by observing the MHD, PDI, *ζ*, and DEE % over time.

### 2.14. Statistical Analysis of the Experiment

The results of the experiments were all determined as mean standard deviation values with statistical analysis performed using a GraphPad Prism^®^ 6 (GraphPad Software Inc., San Diego CA, USA). Data obtained from the MHD, PDI, *ζ*, DEE%, and the in vitro drug release, were subjected to a one-way ANOVA and results were considered statistically significant at *p*-values < 0.05, with a 95% significance level.

## 3. Results and Discussion 

### 3.1. Synthesis and Characterization of StBAclm

**Scheme 1:**
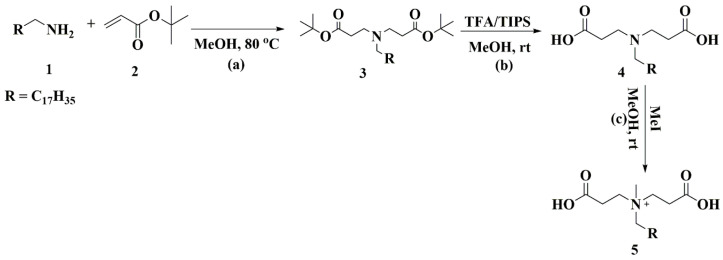
(a). Methanol (MeOH) reflux with constant stirring at 80 °C, 24 h; (b). MeOH at room temperature, 8 h and (c). MeOH, at room temperature, 12 h.

The cationic bicephalic pH-responsive surfactant was synthesized in three steps. The first step involved Michael’s addition reaction, as described in Scheme 1. Compound 3 was synthesized by the dropwise addition of *tert*-butyl acrylate (Compound 2 to Compound 1), and the synthesis was confirmed by ^1^H NMR, ^13^C NMR, and HR-MS, as shown in the Supplementary Material Figure S1. The appearance of a strong single peak at 1.3711 ppm, integrating for 18 protons in ^1^H NMR, and the appearance of carbon peaks at δ 82.1 in ^13^C NMR, representing *tert*-butylate acrylate, confirmed the formation of Compound 3. The second step involved the deprotection of the carboxylic group. Tertiary butyl esters of Compound 3 were deprotected through cleavage to form StBAclm, using TFA/TIPs (Supplementary Material Figure S2). The successful synthesis of StBAclm was confirmed by the disappearance of peaks at 1.3711 in ^1^H NMR and 82 ppm in ^13^C NMR. The third step involved the methylation reaction on the amine group. Methyl iodide was added to Compound 4 to obtain Compound 5. The product was confirmed by the appearance of a single peak at 3.2752 ppm ^1^H NMR and 49.8 ppm in ^13^C NMR and mass spectrometer, respectively (Supplementary Material Figure S3).

### 3.2. Preparation and Characterization of VCM-StBAclm-Qt_1_

#### 3.2.1. Preparation, Characterization and Morphology of VCM-StBAclm-Qt_1_ Quatsomes 

**Scheme 2:**
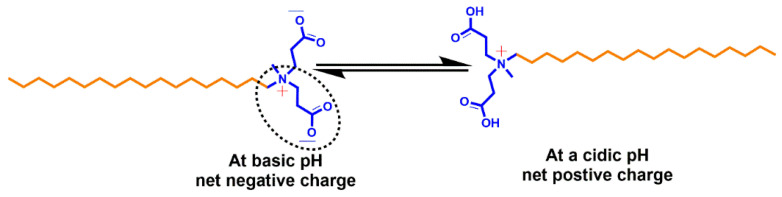
pH response of the bicephalic surfactant and surface charge change.

**Figure 1:**
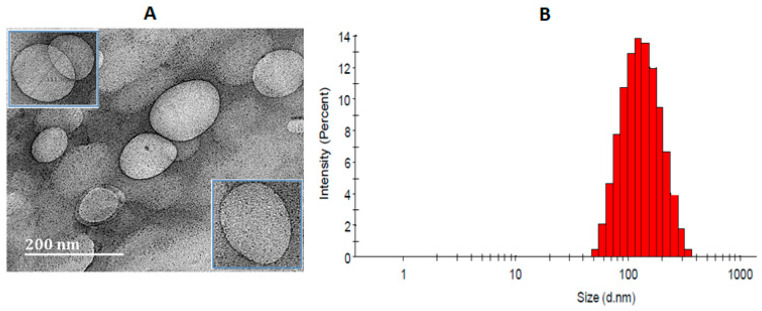
(**A**) Morphology of VCM-StBAclm-Qt_1_ quatsomes scale bar: 200 nm and (**B**) size distribution of VCM-StBAclm-Qt_1_ quatsomes measured using dynamic light scattering (DLS).

Following the successful synthesis of the StBAclm surfactant and the confirmation of its biosafety in in vitro studies, its potential to formulate VCM-StBAclm-Qt_1_ quatsomes was then investigated. Preliminary studies involving different molar ratios of StBAclm: CHol were screened in order to get an optimal formulation of the quatsomes, as shown in Table S1 in the supplementary material. An increase was observed in the concentration above ration 1:1; the particles sizes and PDI increased. It has been reported before that the stabilizing effect of surfactant decreases at high concentrations as the surfactant molecules tend to aggregate in to micelles rather than associating with lipidic molecules [52,63]. The optimized drug-loaded quatsomes (VCM-StBAclm-Qt_1_) had MHD, and PDI of 122.9 ± 3.78 nm and 0.169 ± 0.02, respectively, at physiological pH 7.4 (Figure 1B). The MHD values were similar to the sizes of quatsomes that have been previously reported [13,22]. In contrast, the VCM-StBAclm-Qt_1_ quatsomes in an acid environment of pH 6.0 showed an increase in MHD value from 122.9 ± 3.78 nm to 145.7 ± 5.08 nm. The VCM-StBAclm-Qt_1_ quatsomes demonstrated surface charge switching (*ζ*) from negative (−5.74 ± 2.57 mV) in physiological pH 7.4 to positive (+16.0 ± 1.59 mV) in an acidic environment of pH 6.0 (Table 1). The size of drug delivery vesicles (blank quatsomes) at pH 6.0 and 7.4 were 122.9 ± 3.78 nm and 145.7 ± 5.08 nm, and the charge status revealed pH-responsiveness. The charge at pH 7.4 could be attributed to the bicephalic surfactant that has an overall negative charge at basic pH, where the deprotonated two carboxylic arms cancel out the quartanary ammonium charge. The charge switches to positive at the acidic medium as the carboxylic functional groups are protonated leaving the quarternary ammonium charge positive charge-dominant (Scheme 2).

The DEE% of the VCM-StBAclm-Qt_1_ quatsomes was 52.22 ± 8.4, which is higher compared to other previously reported formulated VCM vesicle systems with DLC% of 13.20 ± 1.17% [64,65]. This could be due to the ability of the VCM to partition, both in the lipidic bilayer and aqueous lumen of the quatsomes. Furthermore, the DSC studies revealed that the lyophilized quatsomes showed the disappearance of the VCM thermal peak when compared to the physical mixture of the excipients and VCM, thus indicating a successful encapsulation of VCM in the quatsomes. It should be noted that after ultrafiltration, the concentration of the StBAclm-Qt in the filtrate was not determined. The VCM-StBAclm-Qt_1_ quatsome morphology, as shown in Figure 1A, was observed to be spherical vesicles, containing a thin membrane and an aqueous core, having a similar MHD size, obtained from dynamic light scattering measurement; and this image is similar to other reported quatsomes [13,66].

#### 3.2.2. Effect of Change in pH on MHD and *ζ*

**Figure 2:**
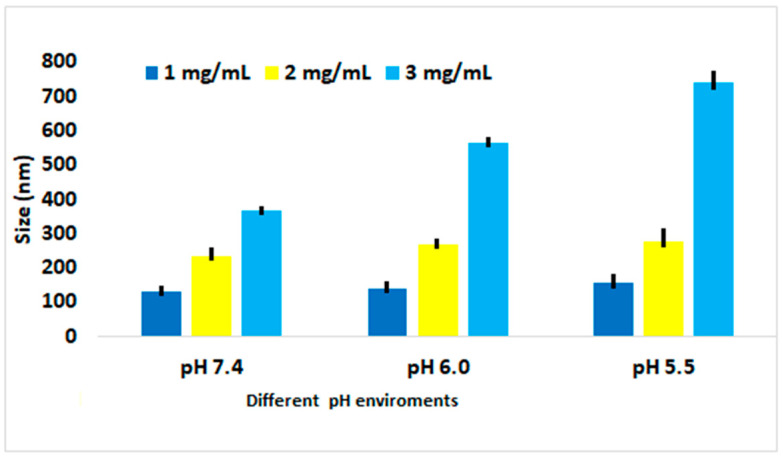
Preliminary, optimized characterization of quatsomes. Effect of the surfactant (StBAclm) concentration on the size of the quatsomes at different pH (n = 3 for all observations).

The effect of pH on size and surface charge-switching (*ζ*) was determined in different pHs, as shown in Figure 2. There was an increase in the size of the quatsomes with a decrease in pH. The effect of pH on the ζ demonstrated surface charge switching from negative at physiological pH 7.4; to positive at acidic pH 6.0 and pH 5.5. The charge switch could be attributed to protonation and deprotonation of the surfactant. At physiological pH, the bicephalic carboxylic groups deprotonated, which neutralized the quaternary charge, leaving the surfactant with an overall negative charge. At acidic pH, the carboxylic groups protonated, gaining a neutral charge; thus the surfactant gained an overall positive charge due to the positive charge on the quaternary nitrogen. Similar *ζ* were reported for liposomes formulated with stearylamine and diacetyl phosphate (DCP), a quaternary compound [67]. Interestingly, the PDI of the system did not significantly change with the change in pH (Table 1). This could be attributed to high stability associated with quatsome formulations [13].

#### 3.2.3. Vancomycin, CHol, and StBAclm Complex and Self-Assembly of Inserted Complexes 

The total binding-free energy was calculated to gain insight into the binding energetics of Vancomycin, cholesterol, and surfactant complex. The MM-GBSA program in AMBER was used in calculating the binding-free energies by extracting snapshots from the trajectories of the compounds. From Table 2, it was observed that Van der Waals (VdW) energy played a major role in the binding of the molecules.

**Figure 3:**
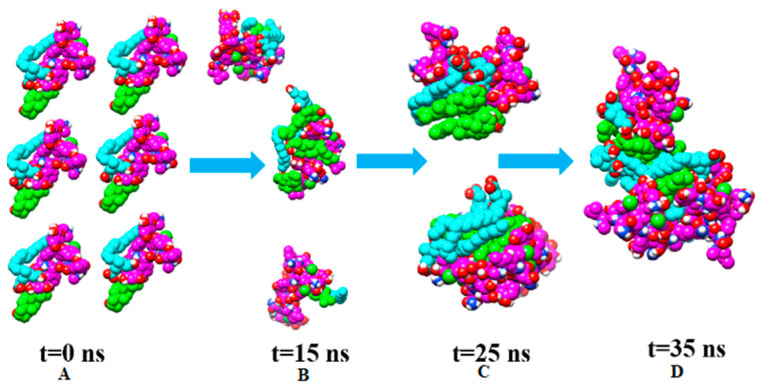
Self-assembly simulation at different points in time (**A**–**D**); where purple is VCM, green is CHol, and sky blue is StBAclm.

The last frame (100 ns) of the first simulation was taken and it was observed that the VCM interacted with the CHol and StBAclm complex. This was replicated in order to make six complexes and perform 35 ns self-assembly simulations. It was observed that these complexes started to assemble within a few nanoseconds (ns) after simulations (Figure 3). Simulation data revealed that at 15 ns three dimers were formed; at 25 ns and 35 ns, two trimers and a hexamer was formed respectively. There was self-assembling of the complexes throughout the simulation period. At 25 ns two trimers were formed with a hydrophilic component on either side of the trimers (Figure 3C). Interestingly, the two trimers fused to form a sort of a bilayer ridge-like structure. The hydrophobic portions at the centre formed non-solvent accessible areas, while the hydrophilic groups were seen on the opposite-facing water (Figure 3D). This could be due to the initial arrangement before assembling into a vesicle. This study indicates the possible steps involved in the formation of the quatsomes.

### 3.3. Cytotoxicity and Hemolysis Assays

#### 3.3.1. In Vitro Cytotoxicity Assay

**Figure 4:**
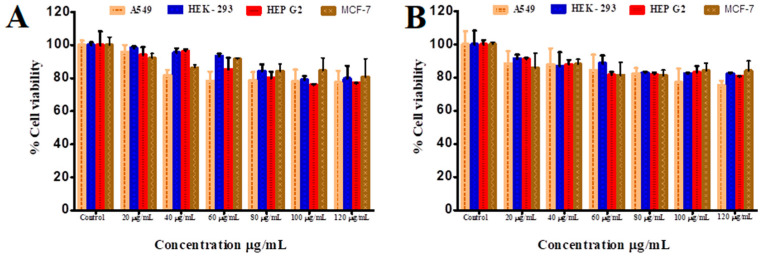
(**A**) Percentage cell viability of StBAclm on different cell lines A-549, HEK-293, Hep-G2, and MCF-7 and (**B**) percentage cell viability of VCM-StBAclm-Qt_1_ quatsomes on different cell lines A-549, HEK-293, Hep-G2, and MCF-7.

The determination of cell viability is a significant assay to evaluate the cytotoxicity of biomaterials and nanosystems [68]. The MTT assay is a rapid quantitative procedure based on the transformation of a yellow tetrazolium salt to insoluble purple formazan crystals in the mitochondria of viable cells [69]. Figure 4A,B shows the bar chart breakdown of the biocompatibility assay of StBAclm and VCM-StBAclm-Qt_1_ quatsomes on four different cell lines (A-549, HEK-293, Hep-G2, and MCF-7 cells). The bicephalic surfactant demonstrated cell viability of 77.31–95.80%, 78.88–98.05%, 75.93–93.90% and 84.11–92.00% against A-549, HEK-293, Hep-G2, and MCF-7, respectively, across all concentrations from 20 to 120 µg/mL. The VCM-StBAclm-Qt_1_ quatsomes demonstrated percentage cell viability, from 77.02 to 88.35% for A-549; 82.25–91.42% for HEK-293; 80.03–91.06% for Hep-G2; and 81.16–85.67 for MCF-7 cell lines across all concentrations of VCM in the quatsomes. Based on the above results, StBAclm and VCM-StBAclm-Qt_1_ quatsomes are considered non-cytotoxic and safe, with greater than 75% cell viability. This result is a preliminary indication of the non-toxic nature of the novel surfactant and nano-system in all the mammalian cells tested [70]. Therefore, based on these results, the drug-loaded quatsomes (VCM-StBAclm-Qt_1_) could be a suitable vehicle for drug delivery.

#### 3.3.2. In Vitro Hemolytic Evaluation of VCM-StBAclm-Qt1 Quatsomes

**Figure 5:**
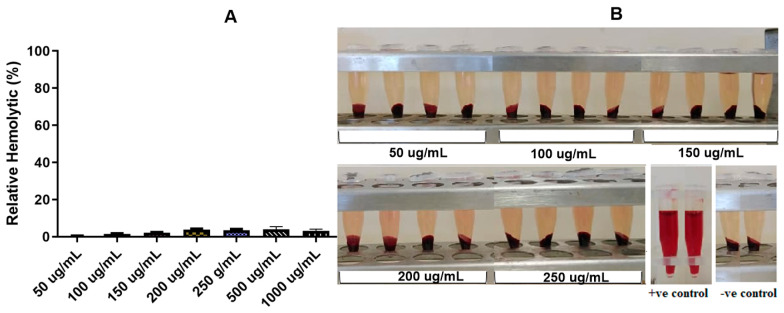
(**A**) Histogram expression of VCM-StBAclm-Qt_1_ in blood using different concentrations as indicated and (**B**). visual observation of the hemolytic activity of VCM-StBAclm-Qt_1_ at different concentrations after centrifugation. The data are the means of four replicates with standard deviations.

One of the challenges in the in vivo application of quaternary surfactants is the hemolysis of red blood cells [71]. To provide additional information about the safety profile and hemocompatibility, StBAclm was evaluated for its hemolytic activity (Figure 5A,B). The results showed that hypotonic distilled water led to erythrocytes rupture, revealing hemolysis. However, there was no hemolysis of the erythrocytes, as observed in the tubes for isotonic saline (PBS) and the VCM-StBAclmQt_1_ quatsomes with 50 to 1000 µg/mL. These results indicated that StBAclm was non- hemolytic in the concentration tested. Higher charge ratios are generally more toxic to red blood cells and a variety of cell types. The non-hemolytic effect of the quatsomes and the quaternary surfactant on the red blood cells could have been due to the overall lower positive charge of the surfactant. The reduction may be due to the neutralizing negative charge and steric hindrance effect of the bispropionic acid heads on the positive charge of the quaternary nitrogen [72,73]. Such a design could result in QAC compounds that can be used systematically with reduced toxic effects.

### 3.4. In Vitro Drug Release Behaviour 

**Figure 6:**
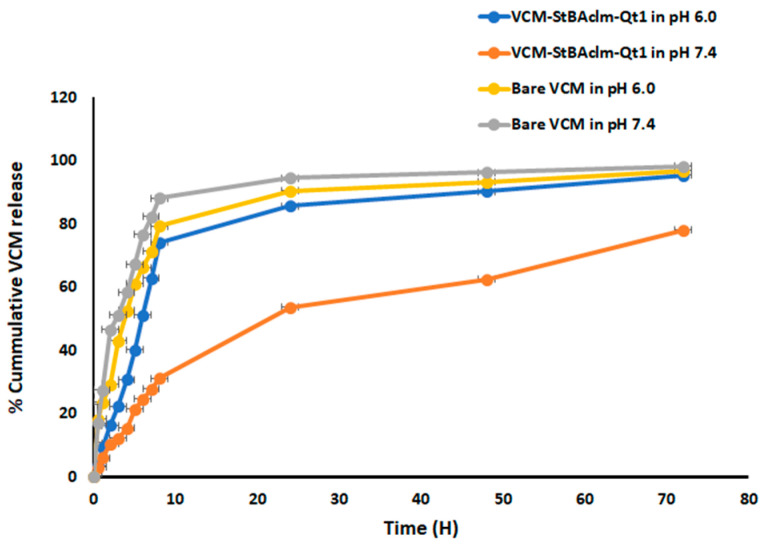
Drug release profiles of bare VCM and VCM-StBAclm-Qt_1_ quatsomes at pH 7.4 and pH 6.0. (mean ± SD, *n* = 3).

The in vitro drug release study was carried out in PBS (pH 6.0 and 7.4) at 37 °C to investigate the cumulative release profile of VCM from the quatsomes. The cumulative amount of VCM released from VCM-StBAclm-Qt_1_ quatsomes was observed to be faster at pH 6.0 compared to pH 7.4 (Figure 6). The higher VCM release at pH 6.0 could be due to the protonation and deprotonation of the amine group in the bicephalic surfactant StBAclm. Protonation of the carboxylic groups in acidic pH might have made the surfactant more hydrophobic, resulting in the rearrangement of the surfactant in the quatsome nanosystem [74,75]. Slow release at pH 7.4, and faster release at pH 6.0, could be beneficial as it could reduce the amount of the drug in general circulation, thus reducing side effects. In addition, it can concentrate the drug in the acid bacterial infection sites, as acidic pH has been found to be in bacterial infection sites, such as respiratory, intra-abdominal, urinary tract, and skin and soft tissue infections [76,77,78]. The release profile during this experiment indicated that VCM release from VCM-loaded StBAclm-Qt_1_ quatsomes was pH-responsive and showed potential for application in targeting drugs to diseases that thrive in an acidic environment.

### 3.5. Stability Studies

The colloidal stability of the quatsomes indicated no significant change in MHD, PDI, *ζ*, and DEE% (*p* > 0.5) over twelve weeks, with formulations stored at pH 7.4 (Table 3). The results are similar to another reported quatsome study by Ferrer-Tasies et al. in 2013, which showed stability with great homogeneity with respect to MHD [13].

### 3.6. In Vitro Antimicrobial Activity

#### 3.6.1. MIC Determination

The antimicrobial activity of StBAclm (the surfactant), the StBAclm-Qt_1_ (blank quatsomes), VCM-loaded quatsomes, and bare VCM were studied against MRSA. The surfactant alone, prior to formulating quatsomes, had MICs of 125 µg/mL and 250 µg/mL in pH 6.0 and 7.4, respectively. The minimum inhibitory concentration (MIC) was very high when compared to other quaternary ammonium compounds (QACs) such as benzalkonium chloride and cetyltrimethylammonium bromide, which have been reported to have MICs in the range of 5–10 µg/mL [79,80] and 0.75–3 µg/mL [81], respectively, across different MRSA strains. The reduced antimicrobial activity of StBAclm, when compared to other QACs, is due to the overall reduced cationic charge density. This reduction could be as a result of the neutralization and steric hindrance effect of bispropionic acid heads [82]. An electrostatic interaction mechanism based on the exchange of counterions between the cationic surface charge and the negatively charged bacterial membrane influences the antimicrobial activity of cationic compounds; the higher the cationic charge ratio the more potent the QAC [82,83]. A lower cationic charge ratio and increased MIC was a trade-off that was envisaged in the design of the novel surfactant in order to increase the safety profile. It is reported that highly cationic compounds have toxic effects and cannot be used systemically as higher cationic charge ratios are generally more toxic to a variety of cell types [25]. The blank quatsomes had MICs that were similar to that of the surfactant alone at both pH. The quatsome consisted of only the surfactant and the cholesterol; the latter did not show any antibacterial activity for MRSA within the concentrations used to formulate quatsomes.

The MICs for the bare VCM at both pH values were constant against MRSA (31.25 µg/mL). However, loading VCM into the quatsomes resulted in MICs of 0.97 µg/mL at acidic pH 6.0 and 3.90 µg/mL at physiological pH 7.4, as shown in Table 4. The effect of loading VCM into the quatsomes resulted in an eight-fold and thirty-two-fold enhanced antimicrobial activity against MRSA at physiological pH 7.4 and acidic pH 6.0, respectively, when compared to bare VCM. Furthermore, the drug-loaded quatsomes at pH 6.0 had a four-fold antibacterial activity enhancement of VCM against MRSA when compared to the activity of the quatsomes at physiological pH 7.4. The results at pH 7.4 were similar to a liposomal formulation produced by Sande et al., who had a four-fold reduction of VCM MIC after encapsulation in a liposome [84]. In another study, encapsulation of VCM in a liposome did not decrease the MIC against MRSA ATCC strain 29213; however, MRSA strain 494 showed a two-fold decrease for the liposome formulation. In the same study, double encapsulation of Cefazolin and VCM resulted in a 7.9 fold reduction in the MIC against the MRSA ATCC 29213 strain [85]. The overall enhanced antibacterial activity by the quatsomes, when compared to the bare drug, could be due to the large surface area to volume ratio due to the smaller size of the vesicles; resulting in the efficient distribution and attachment to the bacteria membrane [52]. Moreover, the surfactant alone and the blank quatsomes had the same MIC, which was similar to the study by Thomas et al [22]. Therefore, the combined antibacterial effects of the surfactant and the loaded VCM in the nanosystem, acting on different sites on the bacteria, might have been the reason for better activity when compared to the bare drug. The literature shows that combination of QAC with other antimicrobial agents results in improved antimicrobial activity of the drug [86,87]. Furthermore, the lipidic nature of the quatsomes could have enhanced the transport of the drug into the thick peptidoglycan layer of MRSA, thus avoiding ion trapping, which is the main resistance mechanism of MRSA to bare VCM [88,89,90], resulting in a lower MIC.

It was noted that drug-loaded quatsomes had better antimicrobial activity at acidic pH than at basic pH. This could be explained by the cationic nature of the surfactant at acidic pH (Scheme 2). The novel surfactant contains a positive charge on the quaternary ammonium nitrogen and bisproprionic acid heads. This leads to the neutralization of the positive charge at basic pH due to deprotonation, thus resulting in a surface charge switch of the quatsomes to negative (Table 1). Moreover, at acidic pH, the quatsomes had faster release of the drug. This might have resulted in increased concentrated release of VCM on the bacterial membrane in lethal concentrations that effectively eliminated the bacteria. These results showed that the use of excipients that respond to pH and have inherent antimicrobial activity could result in a system that can augment the antibacterial activity of loaded antibiotics and efficiently target acidic infection sites where bacteria thrive.

#### 3.6.2. Fractional Inhibitory Concentration (FIC) Studies

Due to the nature of quatsomes, they are known to have antimicrobial activity. To further evaluate the effect of the quatsomes on the antimicrobial activity of the loaded VCM, the fractional inhibitory concentration (FIC) was determined, as shown in Table 5. The impact of the synergistic action of the quatsomes and the VCM was then investigated. The results from the ΣFIC study indicated that there was synergism activity at both pH. The synergistic effect was higher at pH 6 than pH 7.4. This could be due to a higher cationic charge of the quatsomes at pH 6 than 7.4, due to protonation. The synergistic effect could be a result of the combined antimicrobial effect of quatsomes and VCM. The ΣFIC values showed that combining and using materials that have different mechanisms of action to formulate antimicrobial drug delivery systems could greatly improve the efficiency of the antibiotics, at reduced concentrations. This could lead to a reduction in dose-dependent toxicities and side effects [85].

### 3.7. Bactericidal Time Assay of VCM-loaded StBAclm-Qt1 Quatsomes

**Figure 7:**
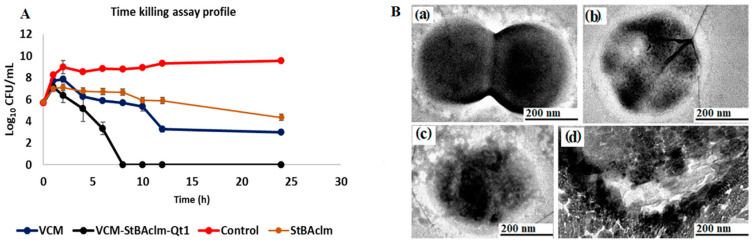
(**A**) Killing kinetics of MRSA exposed to 5 × MIC of VCM-loaded StBAclm-Qt1 quatsomes; VCM; bacteria in PBS 7.4 (positive control); the blank quatsomes (stBAclm); and sterile water (negative control) at different time intervals (n = 3); (**B**) HR-TEM images of (**a**) MRSA treated with VCM-loaded StBAclm-Qt1 quatsomes (5 × MICs), showing the membrane disruption that occurred after (**b**) one hour’s incubation, (**c**) the pores on the bacterial membrane after treatment, and (**d**) the total loss of MRSA cell membrane after four hours of VCM-loaded StBAclm-Qt1 quatsome treatment.

From the antibacterial studies_,_ it was established that the VCM-loaded StBAclm-Qt1 quatsomes could efficiently eliminate MRSA. Therefore, bacterial killing kinetic studies were performed to determine the killing kinetics of bare VCM, VCM-loaded quatsomes, and the blank quatsomes for MRSA. Figure 7A presents the time killing rates of bacteria by the blank quatsomes, VCM, and VCM-loaded StBAclm-Qt1 quatsomes, when exposed to MRSA at 5 × MIC of each treatment over a period of 24 hours’ incubation at 37 °C. The killing kinetics of bare VCM was similar to reports in the literature [91], with a 2.7 log reduction of MRSA. The blank quatsomes had only a 1.34 log reduction in MRSA CFUs over a period of 24 h. The blank quatsomes’ antimicrobial effects were low compared to other antimicrobial quatsomes, where it was reported in the literature to act as an antimicrobial agent on itself; as compared to our system that was designed to deliver a loaded drug for combined effects [22,92]. The VCM-loaded StBAclm-Qt1 quatsomes eliminated almost 100% of MRSA within eight hours of the study. The VCM-loaded quatsome had a 5.7 log reduction of MRSA CFUs in eight hours. Therefore, the improved killing efficacy of the drug-loaded quatsome could be due to the inherent antimicrobial activity of the quatsomes; the penetration enhancement of the lipidic portion of the surfactant [93]; the positive charge that enhances binding to the negatively charged bacterial membrane [94] and the size of the nanosystem, that effectively distributes the bacteria due to its large surface area to volume ratio [52]. The shorter time taken to eliminate MRSA population by VCM-loaded StBAclm-Qt1 quatsomes could be an indication that the quatsome system can be used for a shorter treatment course, compared to bare VCM.

### 3.8. Mechanistic Studies of VCM-loaded StBAclm-Qt1 Quatsomes

#### 3.8.1. Bacterial Membrane Disruption

The effect of VCM-loaded StBAclm-Qt1 quatsomes on the membrane of MRSA was determined by incubating MRSA with our novel VCM-loaded StBAclm-Qt1 quatsomes for approximately five hours. Figure 7B,a shows the perforation of the MRSA cell wall, which demonstrated the effectiveness of the VCM-loaded StBAclm-Qt1 quatsomes’ killing ability (Figure 7B,a–c). Subsequently, after five hours of treatment with the VCM-loaded StBAclm-Qt1 quatsomes, a total loss of bacteria cell wall was observed, demonstrating an effective membrane disruption ability against MRSA, as shown in Figure 7B,d. The interaction of the VCM-loaded StBAclm-Qt1 quatsomes with the MRSA could be due to the antibacterial activity of quaternary bicephalic surfactant, which resulted in the effective delivery of the VCM, leading to bacteria membrane disruption. Quaternary ammonium compounds have long been assumed to disrupt bacterial membranes through electrostatic attraction, followed by intercalation and subsequent disruption [95]. The investigation into how the quatsomes were functionalized revealed that Gram-positive bacteria (MRSA) appear to be affected by the antimicrobial properties of the quatsomes. Additionally, the chemical moieties on the StBAclm carboxylic group affected and disrupted the membranes of bacteria. Furthermore, carboxylic functional groups of the quaternary ammonium disrupt phospholipid lipid bilayers of bacterial membranes by forming pores through various mechanisms called the ‘toroidal pore’ [96]. As the quaternary moieties penetrate the membrane, the head groups of the surfactant are dragged into the lipid tail, resulting in a significant membrane disruption [97]. This result correlates with the MICs and killing kinetics studies, which showed effective killing and clearance of bacteria by the VCM-loaded StBAclm-Qt1 quatsomes. This study revealed the possible mechanism of the quatsomes in the elimination of bacteria.

#### 3.8.2. Fluorescence-Activated Cell Sorting (FACS) Cell Viability

**Figure 8:**
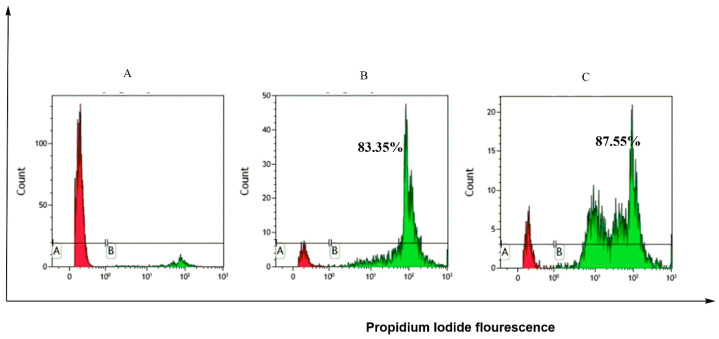
Red represents the gate for live MRSA cells while green represents the gate for dead cells in the population. In the flow cytometry PI emission intensity plot: (**A**) histogram plot of the control group showing live MRSA cells; (**B**) histogram plot of percentage uptake of dead MRSA cells when treated with bare VCM; and (**C**) histogram plot showing percentage uptake of dead MRSA cells when treated with VCM-loaded StBAclm-Qt1 quatsomes. The two peaks of PI fluoresence in (**C**) indicate two populations of bacteria with different extents of cell membrane injury. The second peak represents a bacteria population that had internalised more of the PI, indicating more injury due to drug-loaded quatsome effects, which led to more internalization of the non-cell memberane permeant dye.

The cell viability of MRSA was further evaluated by using fluorescence-activated cell sorting (FACS) techniques and MRSA was incubated with bare VCM and VCM-loaded StBAclm-Qt1 quatsomes at their respective MICs. Under environmental stress conditions, several pathogens enter into a viable, but not cultivable, state. Thus, the plate counts method may overestimate viability efficiency by not detecting reversible, viable-damaged bacterial cells [98]. The MIC study showed that the VCM-loaded StBAclm-Qt1 quatsomes reduced the MIC of VCM by eight-fold. The lowering of the MIC could result in vegetative forms of bacteria, which could give negative results in the plate count method [99]. FACS systems tend to solve this problem by effectively separating the dead cells from viable cells by using cell permeant and non-cell permeant dyes. Figure 8A shows the flow cytometry results of bare VCM and VCM-loaded StBAclm-Qt1 quatsomes. The killing percentage of VCM-loaded StBAclm-Qt1 quatsomes against MRSA cells after incubation was similar to VCM (Figure 7B,a) at their respective MICs, despite having an eight-fold lower concentration. These results demonstrated that, even with the reduction of the MIC of VCM by incorporation into the quatsomes, the system efficiently killed the bacteria without turning the bacteria into a viable, but non-cultivable, state. This state has been attributed to the development of resistant strains when the bacteria are exposed to sublethal concentrations. The lowering of the MIC value of VCM shows the translational potential of this system without compromising the therapeutic effect of the loaded drug. This could lead to a reduction in the dose-dependent side effects of VCM, such as nephrotoxicity and Redman Syndrome [100].

#### 3.8.3. Biofilm Eradication of VCM-Loaded StBAclm-Qt1 Quatsomes using Fluorescence Microscopy

**Figure 9:**
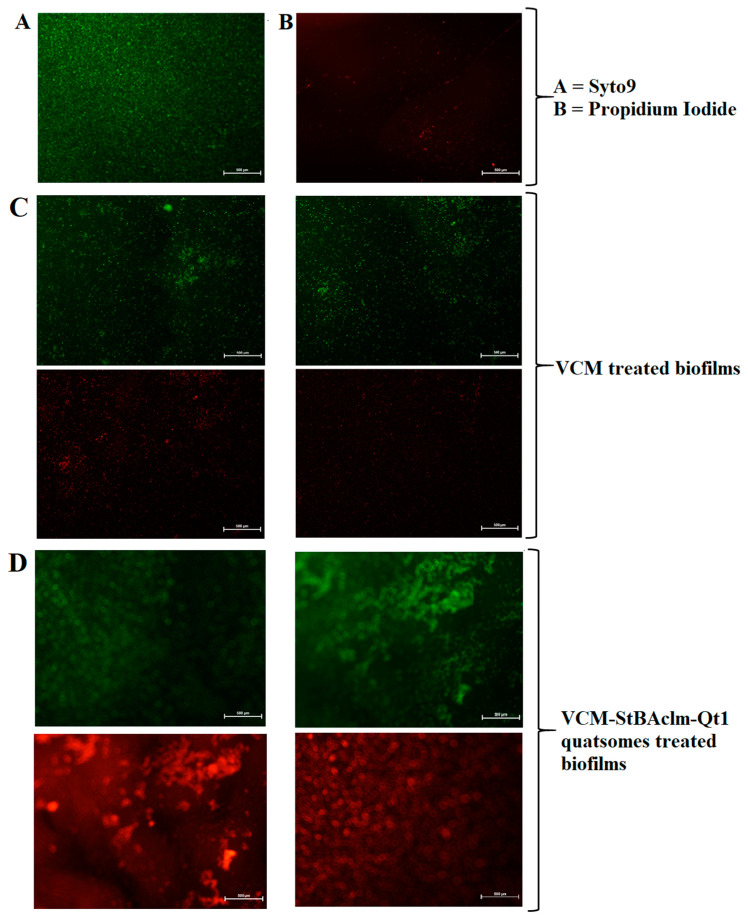
Fluorescence microscopy images of MRSA biofilm: (**A**) Untreated biofilms stained with Syto9. (**B**) Untreated biofilms stained with Syto9 and propidium iodide. (**C**) VCM-treated biofilms. There is more green fluorescence emanating from the Syto9-treated sample than PI-treated biofilms. This could be due to more live cells than dead cells, as PI only penetrates cell membrane-compromised cells. (**D**) VCM-loaded StBAclm-Qt1 quatsomes-treated biofilms, showing increased internalization of PI in the biofilms, as indicated by the red fluorescence. This is an indication of more dead cells in the VCM-loaded StBAclm-Qt1 quatsomes biofilms (scale bar = 500 μm).

Fluorescence microscopy was also used to investigate the quatsomes’ ability to eliminate biofilms. Biofilms are microbial communities attached to surfaces and encased in a protective extracellular polymeric substance (EPS) matrix of microbial origin [101,102]. An EPS matrix acts as a biofilm protective barrier that prevents antimicrobial drugs from penetration, thereby protecting the bacteria cells. A four-days, fully mature MRSA biofilm was grown on a coverslip, treated with bare VCM and VCM-loaded StBAclm-Qt1 quatsomes, and further investigated and analyzed using fluorescence microscopy. Biofilms were stained with 30 µL of PI and Syto9, respectively, diluted up to 1 mL. The samples were kept in the dark for 30 min, followed by washing with sterilized water. Then the coverslips were inverted on the glass slides (Figure 9). Despite the concentration ratio of 1:1, Syto9: PI, the cell permeating dye (Syto9) revealed a high intensity, due to the intact membrane of cells on the coverslip of the untreated samples. However, no fluorescence intensity was observed for PI dye, as it could not penetrate the intact cell membrane and the EPS matrix (Figure 9A). Biofilms treated with bare VCM showed Syto9 intensity and PI fluorescence, demonstrating VCM had a level of penetration into the biofilm-protective EPS matrix, thus reaching the protected MRSA cells (Figure 9B).

When the biofilms were treated with VCM-loaded StBAclm-Qt1 quatsomes, there was an increased red fluorescence due to PI penetration and interaction with the DNA of the MRSA cells (Figure 9C). The higher PI fluorescence emission observed for biofilms treated with VCM-loaded StBAclm-Qt1 indicated that quatsomes could penetrate or destroy the EPS matrix of the biofilm, thus reaching MRSA cells. Upon reaching the MRSA cells, the membrane of the cells was destroyed, resulting in the intercalation of PI with the DNA, as observed in an increased PI fluorescence. This indicates that VCM-loaded StBAclm-Qt1 quatsomes disrupted the biofilms and MRSA cell membrane, which led to a higher PI penetration into the DNA. These results, therefore, demonstrated that VCM-loaded StBAclm-Qt1 quatsomes is a system that can potentially eliminate bacteria biofilms from devices such as respirators [103], catheters (central venous, urinary), prosthetic heart valves, orthopaedic devices [104], and surgical and dental implants [105].

### 3.9. Molecular Antibacterial Studies

#### 3.9.1. Bacterial Cell Membrane Permeability in Terms of Relative Electric Conductivity

The MRSA cell membrane is composed of lipids and proteins, and they are important factors for cell membrane integrity, stability, and permeability. Cell membrane disruption causes leakage of cellular substance, which leads to cell death. Disruption of the bacterial cell membrane can also lead to the leakage of intracellular electrolytes, leading to increased conductivity [106]. To explore the interactions between the quatsomes and cell membrane against MRSA, cell electrical conductivity was observed to investigate bacterial cell disruption. The results from the electrical conductivity of VCM-loaded StBAclm-Qt1 quatsomes showed an increase from 0.357 ± 0.02 to 0.487 ± 0.01 mS cm^−1^ when compared to the bare VCM treatment (Table 6 and Figure 7B,d. This corresponds to a 1.36-fold increase in the electrical conductivity when compared to the bare VCM group (*p* < 0.05). This rise in the electrical conductivity could be due to the displacement of the membrane lipids by quatsomes [107,108,109,110]. Maintaining ion homeostasis is paramount for the proper cell function of the bacteria, including solute transport, metabolic regulation, control of turgor pressure, motility of the cell, and energy creation. Changes in the structural integrity of the cell membrane can affect metabolism and lead to cell death [111]. The increase in conductivity displayed in this study implies that the MRSA cell membrane was destroyed. This led to electrolytic leakage, causing an increase in electrical conductivity; thus leading to cell lysis [112].

#### 3.9.2. Leakage of Proteins and VCM-Loaded StBAclm-Qt1 Quatsomes Analysis

**Figure 10:**
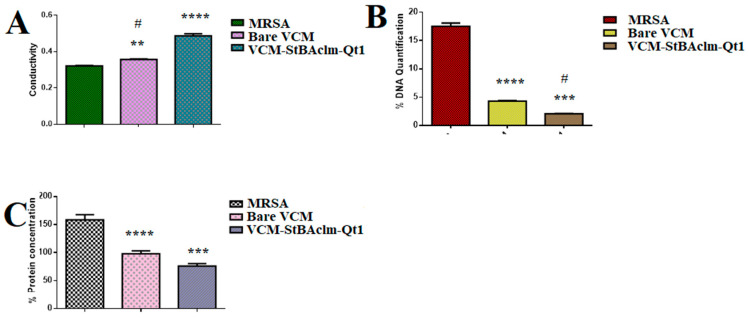
**(A**) Electrical conductivity of bare VCM and VCM-loaded StBAclm-Qt1 quatsomes. ** denotes the statistical significance of bare VCM compared to MRSA (control). **** denotes the statistical significance of bare VCM compared to VCM-loaded StBAclm-Qt1 quatsomes, and # denotes the comparison of VCM-loaded StBAclm-Qt1 quatsomes and MRSA (control); (**B**) reduction of DNA quantification of bare VCM and VCM-loaded StBAclm-Qt1 quatsomes. **** denotes the statistical significance of bare VCM compared to MRSA (control). *** denotes the statistical significance of bare VCM compared to VCM-loaded StBAclm-Qt1 quatsomes, and # denotes the comparison of VCM-loaded StBAclm-Qt1 quatsomes and MRSA (control); (**C**) reduction of protein quantification: bare VCM and VCM-loaded StBAclm-Qt1 quatsomes. **** denotes the statistical significance of bare VCM compared to MRSA (control). *** denotes the statistical significance of bare VCM compared to VCM-loaded StBAclm-Qt1 quatsomes.

Protein plays an essential role in the physiological metabolism of bacteria. The disruption of the membrane results in the loss of membrane integrity, which leads to the leakage of essential proteins responsible for the survival of MRSA cells [113]. As shown in Figure 9B, the DNA concentration of MRSA treated with VCM-loaded StBAclm-Qt1 quatsomes was significantly decreased from 4.3 ± 0.08 to 2.08 ± 0.040 μg·mL^−1^ (a two-fold decrease), compared to bare VCM. The one-way ANOVA test showed significant differences in the protein leakage with a *p*-value < 0.001 in all the groups. The VCM-loaded StBAclm-Qt1 quatsomes might have caused the increase in membrane permeability by destroying the cell membrane, leading to the leakage of a cellular substance. This result showed that VCM-loaded StBAclm-Qt1 quatsomes had a greater impact on the bacterial membrane, which led to DNA leakage (Figure 10) [114]. This implies that, even at low concentrations of VCM in the VCM-StBAclm-Qt_1_, it was more effective compared to bare VCM. Thus, this suggests that the quatsomes can potentially serve as a drug delivery system with effective and low therapeutic dose requirement.

### 3.10. In Vivo Antibacterial Activity

**Figure 11:**
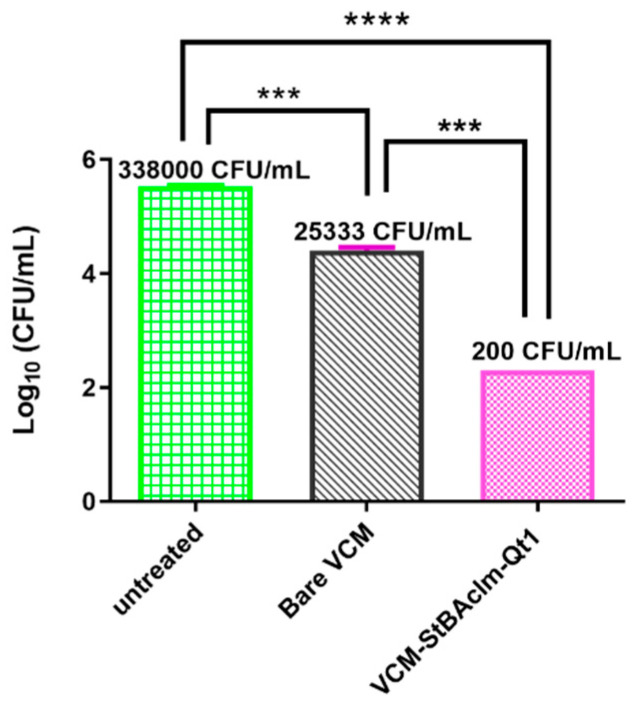
MRSA Log_10_ (CFU/mL) evaluation after 48 h of treatment (mean ± SD; *n* = 3). *** denotes the significant difference between the bare VCM vs. VCM-loaded StBAclm-Qt1 quatsomes and VCM vs. untreated groups. **** denotes the significant difference between VCM-loaded StBAclm-Qt1 quatsomes and the untreated groups.

As proof of the efficacy of VCM-loaded StBAclm-Qt1 quatsomes in animal systems, in vivo studies were conducted using a BALB/c mice skin infection model. The colony-forming unit (CFU) numbers from each treatment group were evaluated and represented as log_10_ (Figure 11). The one-way ANOVA test showed significant differences in the CFUs between all the groups, with a *p*-value = 0.001. The mean MRSA load (log_10_ CFU) obtained from the groups treated with VCM-loaded StBAclm-Qt1 quatsomes and bare VCM, and the untreated groups, were 2.3010 (200 CFU/mL), 4.40 ± 0.05 (25333 ± 3055 CFU/mL), and 5.528 ± 0.014 (338000 ± 11135 CFU/mL), respectively. The mice groups treated with bare VCM had a significantly reduced (126.67-fold) MRSA burden in the mice skin samples when compared to the untreated groups (*p*-value = 0.001). By comparison, and significantly, VCM-loaded StBAclm-Qt1 quatsomes showed a 1690-fold decreased MRSA burden in the skin samples, when compared to the untreated groups. Furthermore, the groups treated with VCM-loaded StBAclm-Qt1 quatsomes had significantly lower (by 13.34-fold) MRSA burdens in the mice skin samples when compared to bare VCM-treated groups. This result is in agreement with other in vivo studies that involved the use of antibiotics such as streptomycin, gentamicin, and doxycycline nanoplexes against bacterial infections [115,116]. The observations from this study revealed the significant potential of VCM quatsomes in combating MRSA infections.

## 4. Conclusions

Due to the diminishing antimicrobial activity of VCM against MRSA, effective and efficient strategies are urgently needed to protect and enhance its antimicrobial activity in order to help combat the widespread infections, globally. In this research, a novel quaternary bicephalic surfactant (StBAclm) was synthesized and used in formulating pH-responsive quatsomes (VCM-StBAclm-Qt_1_) for the delivery and enhancement of VCM against MRSA. The StBAclm characterization was achieved via structural elucidation and in vitro biosafety techniques. The in vitro hemolytic studies confirmed the VCM-loaded StBAclm-Qt1 quatsomes to be non-hemolytic in the concentrations tested. The VCM-loaded StBAclm-Qt1 quatsomes were shown to have smaller MHD, and higher DEE and DLC%; and a pH responsive release with a fast and sustained release of VCM at the acidic environment of pH 6.0, compared to physiological pH 7.4. The molecular modeling simulation (MDS) studies demonstrated the possible self-assembly formation of the quatsomes. In vitro antibacterial studies against MRSA revealed the enhanced antimicrobial activity of the drug-loaded quatsomes, when compared to bare VCM. The superior results of the drug-loaded quatsomes, when compared to the bare drug from the in vitro antimicrobial activity, were further supported by flow cytometry, time-killing assay and biofilms studies. These all showed the better antimicrobial effect of the drug-loaded quatsomes, at eight-fold lower concentrations, when compared to bare VCM. The drug-loaded quatsomes also displayed the ability to eliminate MRSA biofilms. The in vivo studies using the BALB/c mice infected model revealed that the treatment of MRSA infections with VCM-loaded StBAclm-Qt1 quatsomes significantly decreased the MRSA burden, compared to the treatment with bare VCM. The pH-responsiveness, biosafety, and antimicrobial activity of the novel pH-responsive VCM-loaded StBAclm-Qt1 quatsomes indicated their potential for application as a nanodrug carrier for antibiotics.

## Figures and Tables

**Table 1:** MHD, PDI, *ζ*, DEE%, and DLC% of initial screening VCM-StAclm-Qt quatsomes.

CHol: StBAclm (mg/mL)	pH	MHD (nm)	PDI	*ζ* (mV)	DEE%	DLC%
**VCM loaded StAclm-Qt_1_**	7.4	122.9 ± 3.78	0.169 ± 0.02	−5.74 ± 2.57		
	6.0	130.7 ± 5.13	0.201 ± 0.04	+9.89 ± 0.68	52.22 ± 8.4	13.20 ± 1.17%
	5.5	145.7 ± 5.08	0.216 ± 0.04	+16.0 ± 1.59		
**StBAclm-Qt blank**	7.4	139.5 ± 4.506	0.064 ± 0.024	−9.750 ± 3.020		
	6.0	157.4 ± 16.58	0.066 ± 0.015	−6.662 ± 0.439		
	5.5	155.3 ± 2.85	0.175 ± 0.01	+6.01 ± 0.987		

**Table 2:** The binding energies (kcal/mol) for the VCM-CHol-StBAclm computed from the MD trajectories. Affinity energy values and energy components. Van der Waals contributions (ΔEvdW); electrostatic contributions (ΔEele); Change in free energy in gas (ΔGgas) and solvent phase (ΔGsolv) of the process of protonation; Binding free energy (ΔG bind).

Energy Components (kcal/mol)					
Complex	ΔE_vdW_	ΔE_elec_	ΔG_gas_	ΔG_solv_	ΔG_bind_
VCM	−26.44 ± 0.35	32.98 ± 0.79	6.53 ± 0.76	−26.81 ± 0.76	−20.27 ± 0.31
CHol	−15.66 ± 0.30	−0.91 ± 0.19	−16.58 ± 0.34	5.02 ± 0.17	−11.55 ±0.28
StBAclm	−15.06 ± 0.50	33.01 ± 0.83	17.94 ±0.99	−30.28 ± 0.83	−12.33 ± 0.42

**Table 3:** Stability observation of MHD, PDI, ***ζ,*** and DEE % of VCM-loaded StBAclm-Qt_1_ quatsomes after twelve weeks of storage.

	Room Temperature (25 ± 1 °C)				Room Temperature (4 ± 1 °C)			
Time (days)	MHD	PDI	*ζ* (mV)	Content %	MHD	PDI	*ζ* (mV)	Content %
**0**	141.9 ± 3.78	0.285 ± 0.04	−5.74 ± 2.57	100 ± 7.66	141.9 ± 3.78	0.285 ± 0.04	−5.74 ± 2.57	100 ± 7.66
**30**	145.2 ± 5.12	0.265 ± 1.21	−6.12 ± 1.23	98.12 ± 9.9	144.3 ± 2.44	0.242 ± 0.01	−7.29 ± 1.45	99.69 ± 8.27
**60**	143.4 ± 6.77	0.275 ± 2.41	−5.86 ± 2.41	98.91 ± 12.3	140.2 ± 3.21	0.251 ± 0.03	−6.12 ± 1.25	98.87 ± 8.27
**90**	144.1 ± 6.92	0.264 ± 1.03	−5.88 ± 3.10	99.59 ± 11.9	142.3 ± 2.22	0.263 ± 1.02	−6.12 ± 1.25	98.12 ± 5.67

**Table 4:** Minimum inhibitory concentrations (MICs) values of bare VCM, StBAclm-Qt_1_, and VCM-loaded StBAclm-Qt1 quatsomes against MRSA at pH 6.0 and pH 7.4.

	In Vitro Antibacterial Activity at pH 6.0			In Vitro Antibacterial Activity at pH 7.4		
Time (h)	24	48	72	24	48	72
	MRSA (MIC µg/mL)			MRSA (MIC µg/mL)		
Bare VCM	31.25	31.25	31.25	31.25	31.25	31.25
StBAclm blank quatsome	125	125	125	250	250	250
StBAclm surfactant	125	125	125	250	250	250
VCM loaded StBAclm-Qt_1_	0.97	0.97	0.97	3.90	3.90	3.90

All values are expressed as mean ± SD (*n* = 3).

**Table 5:** ΣFractional inhibitory concentration (ΣFIC) of bare VCM and VCM-loaded quatsomes against MRSA at pH 6.0 and 7.4.

**(MRSA) pH 6.0**				
**Duration**	**FIC agent A**	**FIC agent B**	**Σ FIC (agent A + agent B)**	**Interpretation**
24 h	0.031	0.0077	0.0387	Synergy
48 h	0.031	0.0077	0.0387	Synergy
72 h	0.031	0.0077	0.0387	Synergy
**(MRSA) pH 7.4**				
**Duration**	**FIC agent A**	**FIC agent B**	**Σ FIC (agent A + agent B)**	**Interpretation**
24 h	0.125	0.015	0.140	Synergy
48 h	0.125	0.015	0.140	Synergy
72 h	0.125	0.015	0.140	Synergy

**Table 6:** The analysis of MRSA cell membrane permeability after treatment with bare VCM and VCM-loaded StBAclm-Qt1 quatsomes.

Parameters	Control	Bare VCM	VCM-StBAcm-Qt_1_ Quatsomes
Electrical conductivity (mS cm^−1^)	0.321 ± 0.01	0.357 ± 0.02	0.487 ± 0.01
DNA concentration (μg·mL^−1^)	17.0 ± 0.490	4.3 ± 0.08	2.08 ± 0.040
Protein concentration (μg·mL^−1^)	158.58 ± 8.54	98.12 ± 4.88	75.94 ± 4.10

Values are expressed as mean ± SD.

## Supplementary Materials

The following are available online at https://www.mdpi.com/1999-4923/12/11/1093/s1, Scheme S1: (a). Dichloromethane (DCM), reflux with constant stirring at 80 °C, 24 h, (b). DCM, room temperature, 8 h and (c). DCM, room temperature, 12 h, Figure S1: ^1^H NMR of StBA, Figure S2: ^13^C NMR of StBA, Figure S3: FT-IR of StBA, Figure S4: HR-MS (ES-TOF): [M + H] ^+^ for StBA, Figure S5: ^1^H NMR of StBAcl, Figure S6: ^13^C NMR of StBAc, Figure S7: FT-IR of StBAcl, Figure S8: HR-MS (ES-TOF): [M + H] ^+^ for StBAcl, Figure S9: ^1^H NMR of StBAclm, Figure S10: ^13^C NMR of StBAclm, Figure S11: FT- IT of StBAclm, Figure S12: HR-MS (ES-TOF): [M + H] ^+^ for StBAclm, Figure S13: DSC thermographs A) VCM; B) StBAclm; C) CHol; D) Physical mixture (VCM, StBAclm and CHol); D) Lyophilized VCM-StBAclm-Qt_1_ quatsomes, Table S1: Particle size, PDI, ZP and EE% of initial screening VCM-StAclm-Qt quatsomes.

## Abbreviations

ATP	Adenosine triphosphatase
A-549	Adenocarcinomic alveolar basal epithelial cells
BCA	Bicinchoninic acid
CDC	Centers for Disease Control and Prevention
CTAB	Cationic hexadecyltrimethylammonium bromide
CFU	Colony-forming unit
CHol	Cholesterol
DCM	Dichloromethane
DLC	Drug loading capacity
DMSO	Dimethyl sulfoxide
DNA	Deoxyribonucleic acids
DSC	Differential scanning calorimetry
DEE	Drug encapsulation efficiency
FT-IR	Fourier transform infrared
HR-MS	High-resolution mass spectrometry
HEK-293	Human embryonic kidney cell lines
Hep-G2	Liver hepatocellular carcinoma cell lines
MCF-7	Human breast adenocarcinoma cell lines
MDT	Mean dissolution time
MHA	Mueller–Hinton agar
MHD	Mean hydrodynamic diameter
MHB	Mueller–Hinton Broth
MI	Methyl iodide
MICs	Minimum inhibitory concentrations
MRSA	Methicillin-resistant *Staphylococcus aureus*
MTT	3-(4,5-dimethylthiazol-2-yl)-2,5-diphenyltetrazolium bromide
NB	Nutrient broth
OD	Optical density
PDI	Polydispersity index
PI	Propidium iodide
pDNA	Plasmid deoxyribonucleic acid
PBS	Phosphate saline buffer
RMSE	Root mean square error
RBC	Red blood cells
R^2^	Correlation coefficient
SA	Stearylamine
siRNA	Small interfering ribonucleic acid
tBA	*Tert*-butyl acrylate
TFA	Trifluoroacetic acid
TIPs	Triisopropylsilane
UVis-Spec	UV Spectrophotometer
VCM	Vancomycin
*ζ*	Zeta potential
^1^H NMR	Proton nuclear magnetic resonance
^13^C NMR	Carbon 13 nuclear magnetic resonance
