# Supplementary material for: Formulation of pH-Responsive Quatsomes from Quaternary Bicephalic Surfactants and Cholesterol for Enhanced Delivery of Vancomycin against Methicillin Resistant Staphylococcus aureus
Source: Pharmaceutics. 2020 Nov 14;12(11):1093. doi: 10.3390/pharmaceutics12111093 (PMC7696852; doi:10.3390/pharmaceutics12111093)
Supplement: Supplementary file 1 [file pharmaceutics-12-01093-s001.pdf]

pharmaceutics

Supplementary Materials: Formulation of pH-Responsive Quatsomes from Quaternary Bicephalic Surfactants and Cholesterol for Enhanced Delivery of Vancomycin against Methicillin Resistant *Staphylococcus aureus*

Daniel Hassan, Calvin A. Omolo, Victoria Oluwaseun Fasiku, Ahmed A Elrashedy, Chunderika Mocktar, Bongani Nkambule, Mahmoud E. S. Soliman and Thirumala Govender

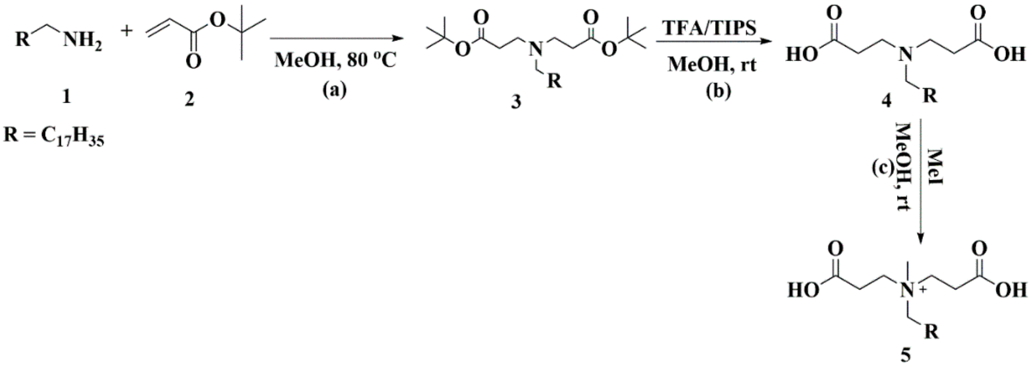

Scheme S1. a). Methanol (MeOH), reflux with constant stirring at 80 °C, 24 h, **b**). DCM, room temperature, 8 h and **c**). DCM, room temperature, 12 h.

S1. Synthesis of Compound 3 (di-*tert*-butyl 3,3'-(octadecylazanediyl)dipropionate

Compound 3 was synthesized following a reported procedure [1]. In brief, *tert*-butyl acrylate (3.5 g; 27.3 mmol) dissolved in methanol (30 mL) and the solution was added to stearyl amine in methanol (3.4 g; 12.6 mmol) (20 mL). The mixture was stirred and refluxed at 80 °C for 24 h and the progress of the reaction was monitored via thin layer chromatography. The crude product was washed with chloroform several times to remove excess *tert*-butyl acrylate (tBA) and the solvent and tBA were removed in vacuo. The crude product was purified via column chromatography (silica, mesh size 60–100) (hexane: ethyl acetate, 7:3) to obtain a white solid (compound 3) (3.7 g; yield 52.67%). FT-IR: 2922, 2852, 1460, 1366, 1252, ^{1}H NMR (400 MHz, $CDCl_3$): 0.7–0.8 (m; 3H), 1.2 (s; 32H), 1.4 (s; 18H), 2.2–2.3 (m; 4H); 2.61–2.7 (m; 2H). ^{13}C NMR (400 MHz, $CDCl_3$): δ 8.4, 14.1, 22.7, 27.4, 29.7, 31.9, 33.7, 49.4, 53.8, 80.2, 91.5, 172.1; HR-MS (Micro-TOF QII): HR-MS (Micro-TOF QII): Mass *m*/*z* calculated for $C_{32}H_{63}NO_4$: 525.48, found 526.56 [M + H] (Figure S1–S4).

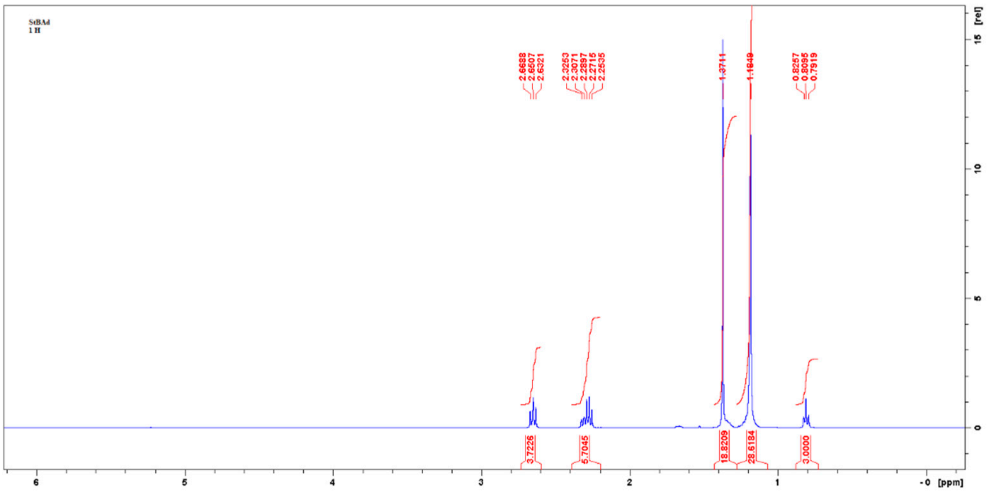

Figure S1. ^{1}H NMR of StBA.

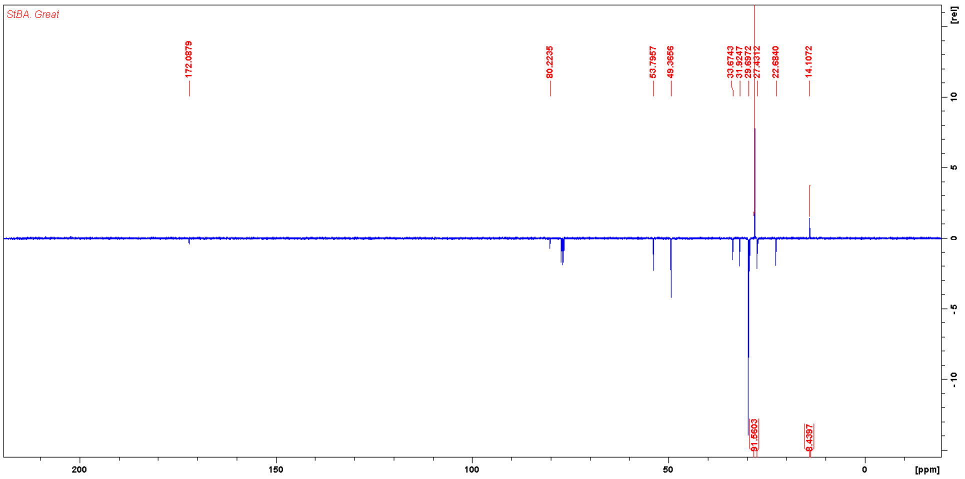

Figure S2. ^{13}C NMR of StBA.

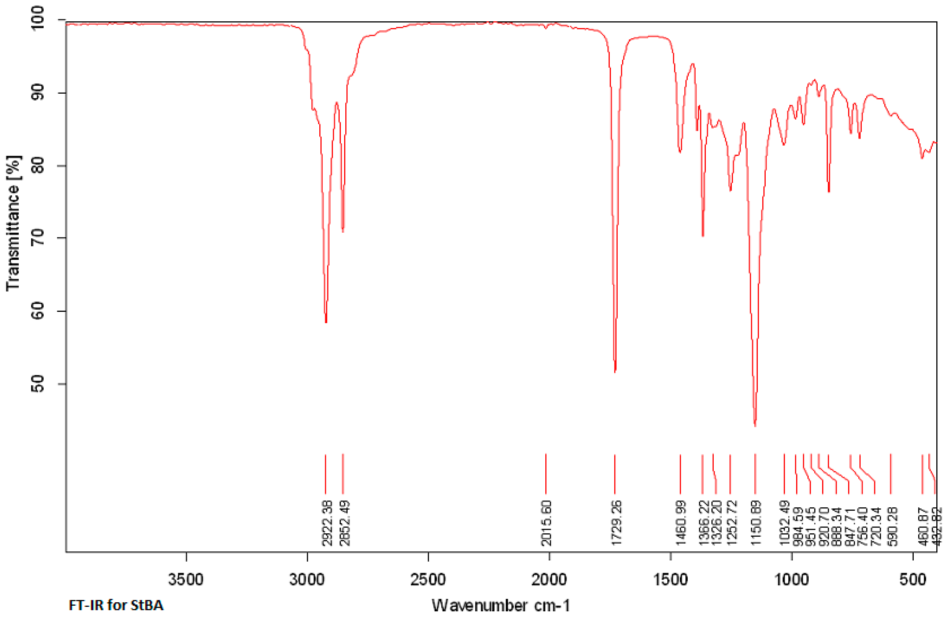

Figure S3. FT-IR of StBA.

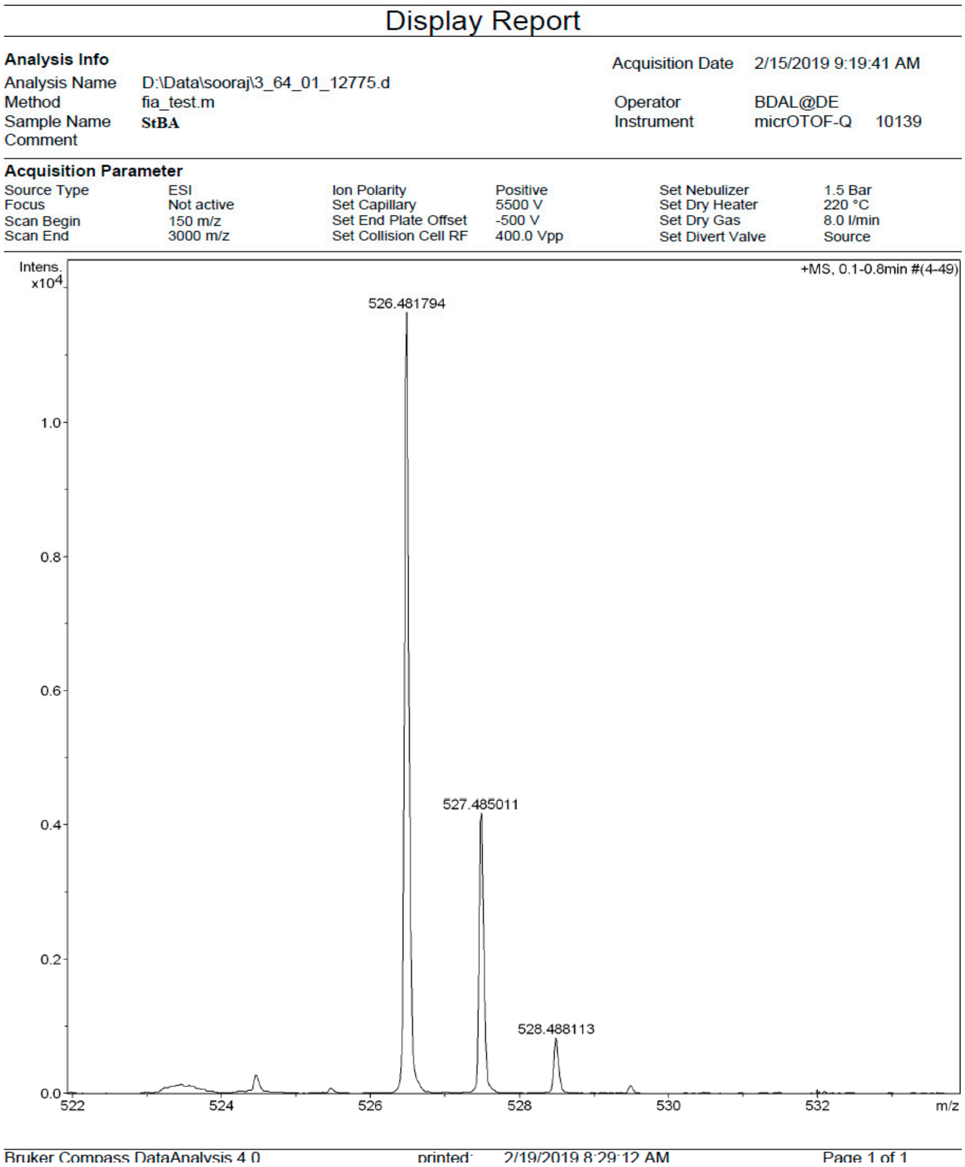

Figure S4. HR-MS (ES-TOF): [M + H]$^+$ for StBA.

S2. Synthesis of Compound 4 (3, 3'-(octadecylazanediyl)dipropionic acid

To a solution of Compound 3 (2.06 g; 0.92 mmol) in methanol (40 mL), trifluoroacetic acid/, triisopropylsilane (TFA/TIPs) (3.63g; 46 mmol) was added at room temperature, as reported in the literature [2]. The resulting mixture was stirred vigorously at room temperature for 6-hours afterward, the solvents and the excess TFA were removed in vacuo and the subsequent residue was properly washed in hexane to obtain a white semi-white compound 4 (StBAcl) (0.56 g; yield 71.70%). FT-IR: 2914,1703, 1436, 1325, 822, cm^{-1}. ^{1}H NMR (400 MHz, DMSO): δ 0.8 (t, 3H), 1.2 (s, 32H), 2.7–2.8 (t, 4H), 3.0–3.1 (t, 2H), 3.3–3.3 (m, 4H). ^{13}C NMR (400 MHz, D_2O): δ 14.4, 22.6, 23.6, 26.4, 28.9, 29.5, 31.8, 39.9, 48.6, 58.2,172.1; HR-MS (Micro-TOF QII): Mass *m/z* calculated for calculated for $C_{24}H_{47}NO_4$: 413.35, found 414.44 [M + H] (Figure S5–S8).

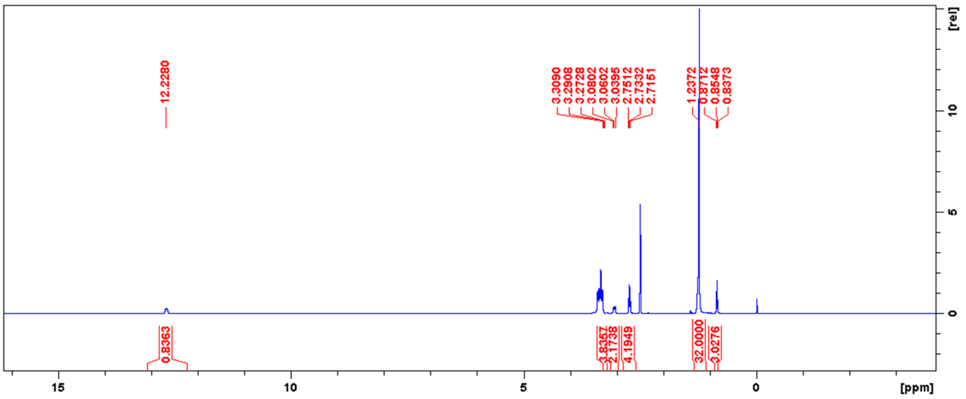

Figure S5. ^{1}H NMR of StBAcl.

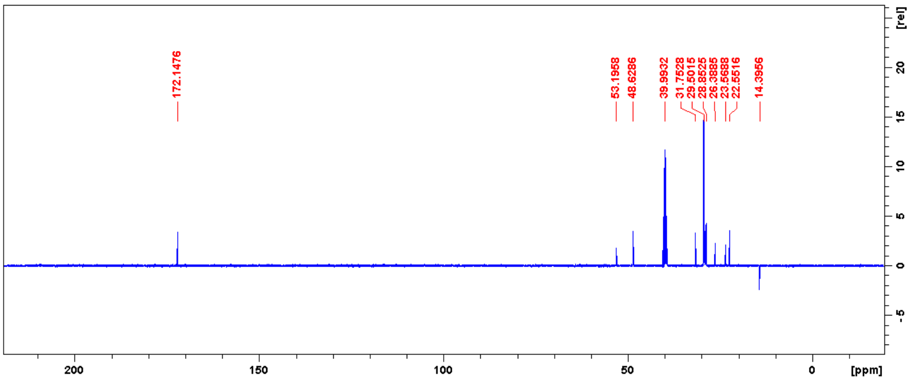

Figure S6. ^{13}C NMR of StBAc.

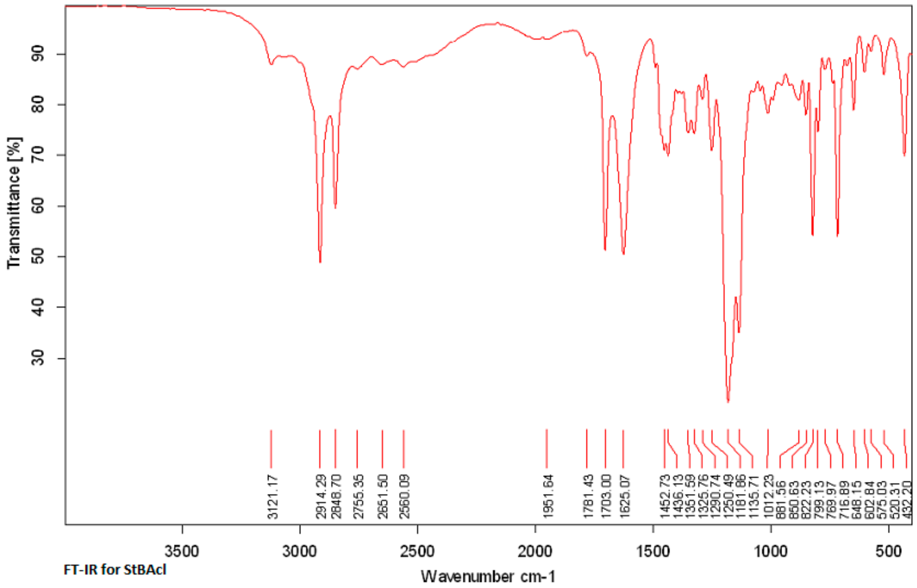

Figure S7. FT-IR of StBAcl.

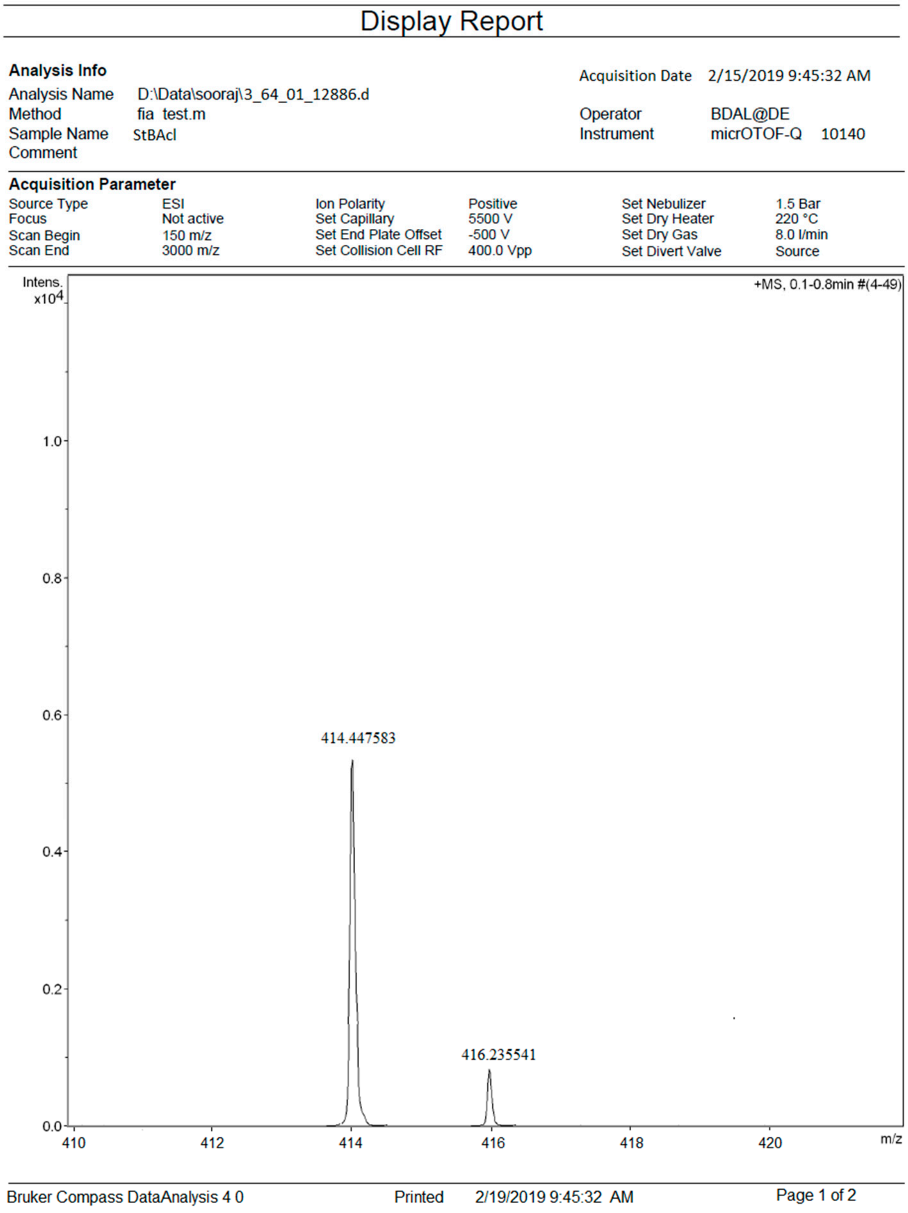

Figure S8. HR-MS (ES-TOF): [M + H]$^+$ for StBAcl.

S3. Synthesis of Compound 5 (*N*, *N*-bis(2-carboxyethyl)-*N*-methyloctadecan-1-aminium

Compound 5 was synthesized following the reported procedure [1]. In summary, compound 5 (2.8 g; 7.24 mmol) was dissolved in methanol; (3.3 g; 23.2 mmol) of iodomethane was added spontaneous (60 mL) at room temperature for 24-hours. After reaction completion, excess methanol and iodomethane were removed in vacuo and the crude product was properly washed in hexane to obtain a yellowish thick oil ((2.2 g; yield 70.9%) compound 5 [3]. FT-IR: 2918, 2850, 1713, 1659, 1468, 1369, 1340, 1153, 942, 843, 798, 719 cm^{-1}. ^{1}H NMR (400 MHz, $CDCl_3$): 0.8–0.82 (t; 3H), 1.2 (s; 32H), 2.7–2.8 (t; 4H); 3.07 - 3.1 (m; 2H), 3.1 (s; 3H), 3.2–3.4 (m;4H). ^{13}C NMR (400 MHz, $CDCl_3$): δ 13.0, 22.3, 23.5, 26.1, 27.9, 28.8, 29.1, 29.1, 29.2, 29.3, 29.4, 31.7, 47.2, 47.4, 47.6, 47.8, 48.0, 49.3, 53.6, 172.7; HR-MS (Micro-TOF QII): Mass *m*/*z* calculated for calculated for $C_{25}H_{50}$ NO_4^+: 428.37, found 429.55 [M + H] (Figure S9–S12).

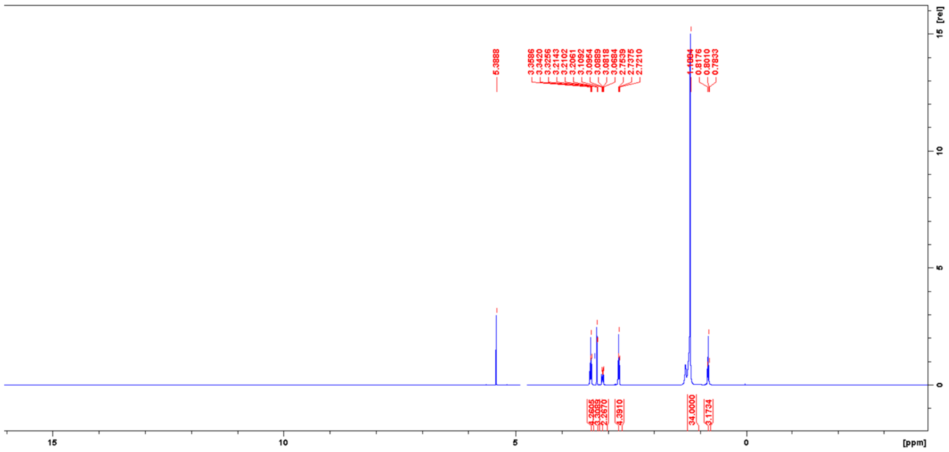

Figure S9. ^{1}H NMR of StBAclm.

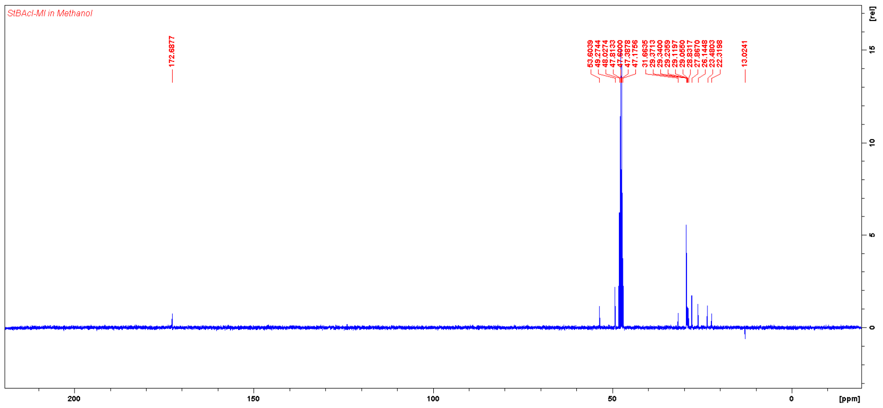

Figure S10. ^{13}C NMR of StBAclm.

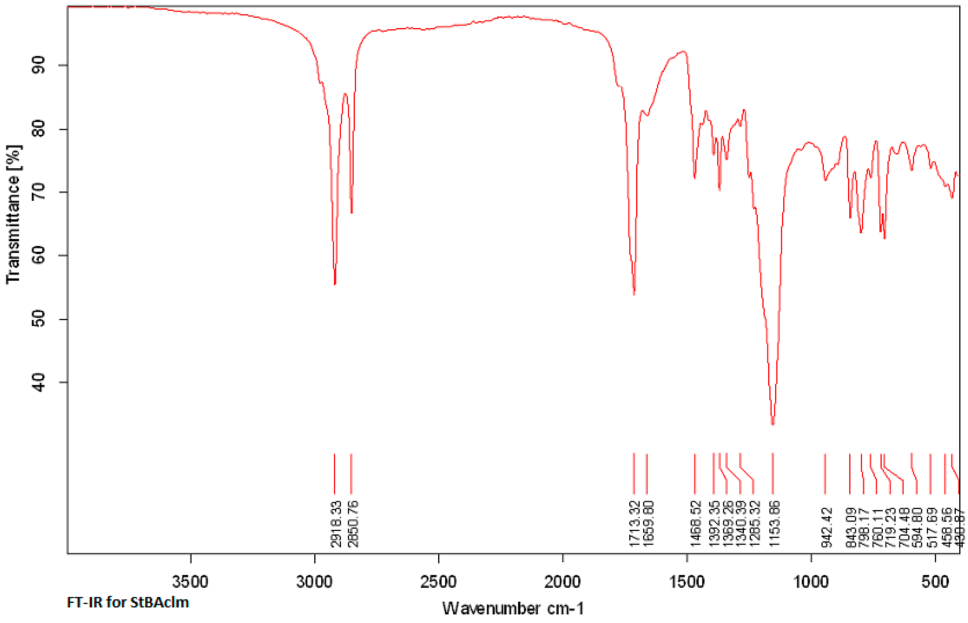

Figure S11. FT- IT of StBAclm.

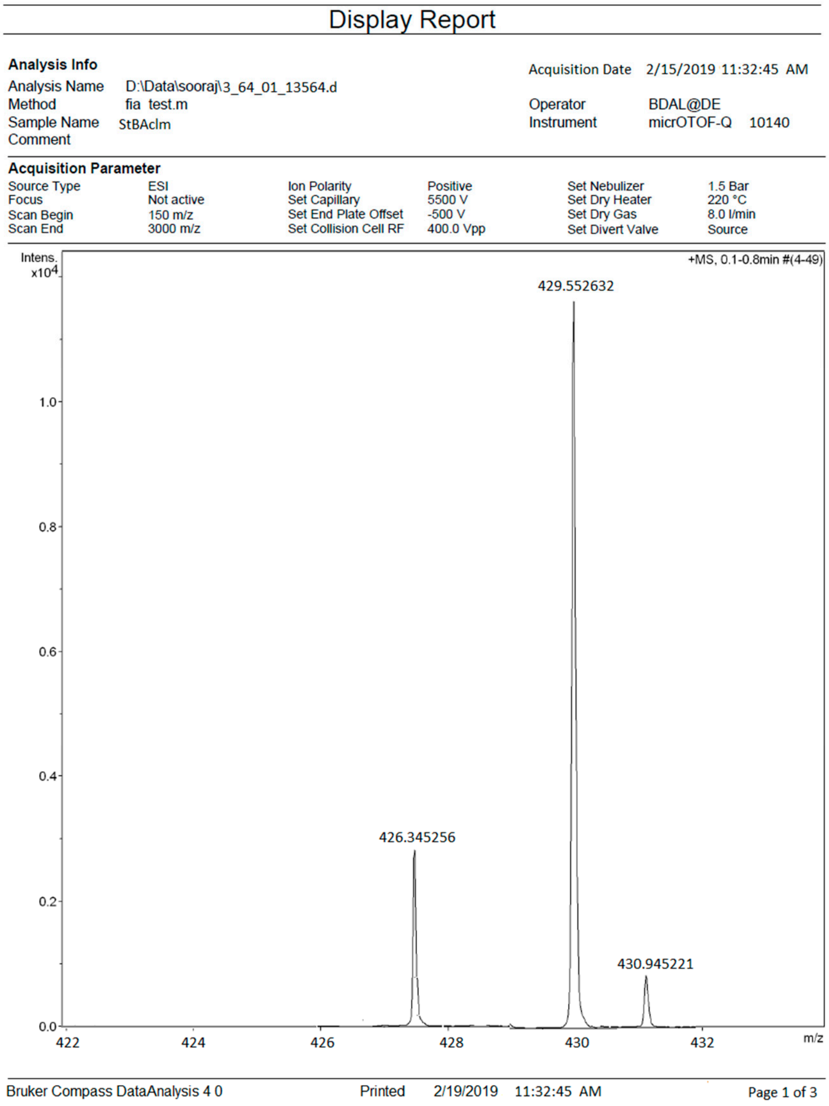

Figure S12. HR-MS (ES-TOF): $[M + H]^+$ for StBAclm.

Table S1. Particle size, PDI, ZP and EE% of initial screening VCM-StAclm-Qt quatsomes.

Chol: StBAclm (mg/mL)	pH	Size (nm)	PDI	ZP (mV)	EE%
	7.4	122.9 ± 3.78	0.169 ± 0.02	-5.74 ± 2.57	
1:1 (VCM-StAclm-Qt_1)	6.0	130.7 ± 5.13	0.201 ± 0.04	+9.89 ± 0.68	52.22±8.4
	5.5	145.7 ± 5.08	0.216 ± 0.04	+16.0 ± 1.59	
	7.4	234.1 ± 58.7	0.246 ± 0.08	-8.65 ± 9.99	
1:2 (VCM-StAclm-Qt_2)	6.0	269.2 ± 35.7	0.355 ± 0.09	+13.1 ± 1.44	46.77 ± 0.86
	5.5	277.2 ± 9.05	0.324 ± 0.09	+13.9 ± 4.08	
	7.4	367.4 ± 16.9	0.441 ± 0.09	-8.30 ± 5.35	
1:3 (VCM-StAclm-Qt_3)	6.0	562.5 ± 65.6	0.449 ± 0.09	+10.3 ± 1.24	50.75 ± 2.85
	5.5	740.5 ± 110	0.657 ± 0.05	+13.6 ± 1.82	

S4. Thermal profile using Differential scanning calorimetry (DSC)

The thermographs for quatsomes and their constituents were shown in Figure S13 were determined by DSC (Shimadzu DSC-60, Tokyo Japan). VCM, StBAclm, CHol, physical mixture (VCM, StBAclm and CHol) and the lyophilised VCM-StBAclm-Qt_1 quatsomes thermoprofiles displayed their distinctive endothermic melting points at 126.97 °C, 170.05 °C, 155.68 °C, and (129.49 °C, 151.18 °C and 199.08 °C) respectively. In the physical mixture, showed the respective peaks of all

the ingredients used to formulate the quatsome. In the lyophilised formulation, the thermal peaks of the VCM disappeared, which could indicate the encapsulation of VCM. Furthermore, such disappearance of the VCM peaks might be due to the conversion of the crystalline form of VCM molecules into an amorphous form.

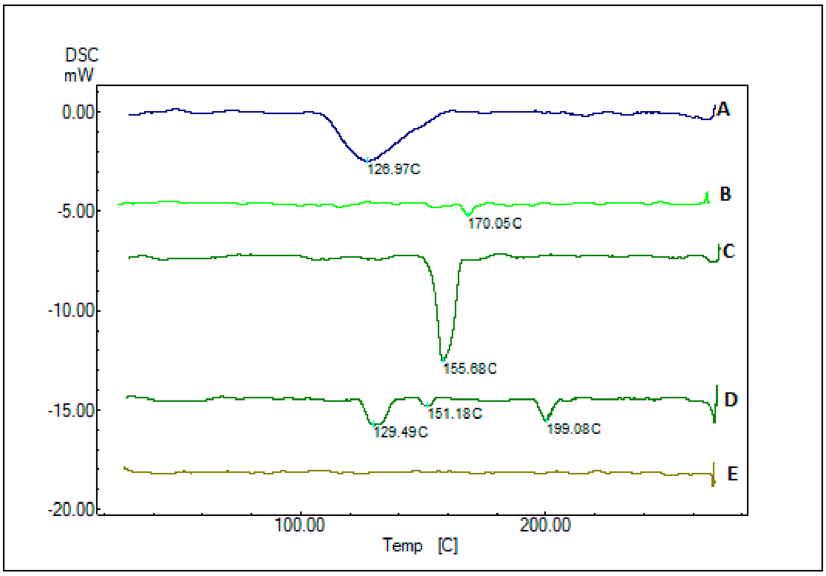

Figure S13. DSC thermographs **A**) VCM; **B**) StBAclm; **C**) CHol; **D**) Physical mixture (VCM, StBAclm and CHol); **D**) Lyophilized VCM-StBAclm-Qt_1 quatsomes.

S5. Drug entrapment efficiency (DEE %) and drug loading capacity (DLC%)

The VCM-StBAclm-Qt quatsomes were further characterized to determine DEE% and DLC%. The DEE% was determined by an ultrafiltration method, as previously reported [4,5]. Briefly, exactly, 2 mL of the drug-loaded quatsomes was loaded into the Amicon® Ultra-4 centrifugal filter tubes (Millipore Corp., USA) having 10 kDa pore size and centrifuged at 3000 rpm for exactly 30 min at 25 °C. The amount of the unentrapped drug in the filtrate was determined spectrophotometrically using Shimadzu UV 1601, Japan at 280 nm. The regression equation of the calibration curve (y = 0.0038x − 0.0031), with a linear regression coefficient (R^2) of 0.9998, was used to determine the unknown drug concentration values. The experiment was done in triplicate, and the DEE% was calculated using the equation below. The DLC% was determined by freeze-drying the quatsomes, and the weight of VCM was calculated using the Equation (1) and Equation (2) below.

$$\text{DEE \%} = \left(\frac{\text{Weight of VCM in quatsomes}}{\text{Weight of VCM added during preparation}}\right) \times 100 \quad (1)$$

$$\text{DLC \%} = \left(\frac{\text{Weight of VCM in quatsomes}}{\text{Total weight of quatsomes}}\right) \times 100 \quad (2)$$

S6. Fractional inhibitory concentration (FIC)

The combined antimicrobial effect of blank quatsome and VCM in the VCM StBAclm-Qt quatsomes against MRSA were assessed using the FIC method [6]. The method is based on the Loewe additivity zero-interaction theory. The theory states that a drug cannot interact with itself and if the effect of combinations are synergistic less of the drug is needed for activity. On the other hand, when antagonism occurs, more of the drug would be needed to produce the same effect as the drug alone. The FIC index was calculated using the Equation (3) below:

$$\Sigma \text{ FIC} = \text{FIC of agent A (Bare VCM)} + \text{FIC of agent B (Blank Quatsomes)} \quad (3)$$

FIC of agent A = MIC of agent A in combination with agent B/ MIC of agent A alone, FIC of agent B = MIC of agent B in combination with agent A/ MIC of agent B alone

Interpretation:

Synergy is defined as **Σ FIC** ≤ 0.5

Indifference is defined as 0.5 < **Σ FIC** ≤ 4

Antagonism is defined as **Σ FIC** > 4

Additive is defined as > 0.5 **Σ FIC ≤** 1

Agent A: (Bare VCM)

Agent B: (StBAclm)

Agents (A and B) in combination: (VCM-StBAclm-Qt_1)

S7. Downstream physiological phenomena: electrical conductivity quantification

1% MRSA was inoculated in 10 mL MHB with bacteria (control), bare VCM and VCM-StBAclm-Qt quatsomes (100 μg/mL) and cultured for 16-hours. 3 mL of the cultured samples were centrifuged at 4000 rpm for 15-min and the supernatant was collected in order to determine its electrical conductivity [7]. The electrical conductivity was determined using an electrical conductivity meter (OHAUS USA) at pH 7.4 and calculated using the equation below (n = 3). Where Q refers to conductivity change rate, Qs and Qc are the conductivity of the test groups and the control group respectively (Equation (4)).

$$\mathrm{Q}\,\% = \left(\frac{\mathrm{Qs} - \mathrm{Qc}}{\mathrm{Qc}}\right) \times\ 100\,\% \quad (4)$$

S8. Determination of DNA and protein quantification

The samples were incubated with the bacteria and centrifuged according to the procedure in Section 7 above The DNA concentration of MRSA treated with bare VCM and VCM-StBAclm-Qt quatsomes were extracted using DNA Kit (Zymo Research, Irvine, CA, USA), and analyzed using Nanodrop 1000 Thermo Fisher Scientific, Waltham, MA, USA at 562 nm absorbance. While the protein concentration of MRSA treated with bare VCM and VCM-StBAclm-Qt quatsomes was determined and analyzed using BCA Kits based on the bicinchoninic acid method on Microplates Spectrophotometer (Spectrostar Nano, Ortenberg, Germany) [7] at an absorbance of 562 nm.

References

1. Soto-Castro, D.; Cruz-Morales, J.A.; Apán, M.T.R.; Guadarrama, P. Synthesis of Non-Cytotoxic Poly(Ester-Amine) Dendrimers as Potential Solubility Enhancers for Drugs: Methotrexate as a Case Study. *Molecules* **2010**, *15*, 8082–8097.
2. Makhathini, S.S.; Kalhapure, R.S.; Jadhav, M.; Waddad, A.Y.; Gannimani, R.; Omolo, C.A.; Rambharose, S.; Mocktar, C.; Govender, T. Novel two-chain fatty acid-based lipids for development of vancomycin pH-responsive liposomes against Staphylococcus aureus and methicillin-resistant Staphylococcus aureus (MRSA). *J. Drug Target.* **2019**, *27*, 1094–1107.
3. Krishna, T.R.; Jayaraman, N. Synthesis of Poly(propyl ether imine) Dendrimers and Evaluation of Their Cytotoxic Properties. *J. Org. Chem.* **2003**, *68*, 9694–9704.

4. Wang, Q.; Jiang, H.; Li, Y.; Chen, W.; Li, H.; Peng, K.; Zhang, Z.; Sun, X. Targeting NF-kB signaling with polymeric hybrid micelles that co-deliver siRNA and dexamethasone for arthritis therapy. *Biomaterials* **2017**, *122*, 10–22.
5. Liu, N.; Han, J.; Zhang, X.; Yang, Y.; Liu, Y.; Wang, Y.; Wu, G. pH-responsive zwitterionic polypeptide as a platform for anti-tumor drug delivery. *Colloids Surfaces B: Biointerfaces* **2016**, *145*, 401–409.
6. Seedat, N.; Kalhapure, R.S.; Mocktar, C.; Vepuri, S.; Jadhav, M.; Soliman, M.; Govender, T. Co-encapsulation of multi-lipids and polymers enhances the performance of vancomycin in lipid–polymer hybrid nanoparticles: In vitro and in silico studies. *Mater. Sci. Eng. C* **2016**, *61*, 616–630.
7. Lin, L.; Mao, X.; Sun, Y.; Cui, H. Antibacterial mechanism of artemisinin / beta-cyclodextrins against methicillin-resistant Staphylococcus aureus (MRSA). *Microb. Pathog.* **2018**, *118*, 66–73.
